# Inducing high exo selectivity in Diels–Alder reaction by dimethylborane substituent: a DFT study

**DOI:** 10.1038/s41598-022-26685-y

**Published:** 2022-12-23

**Authors:** Davood Taherinia, Alireza Fattahi

**Affiliations:** Department of Chemistry, Sharif University of Technology, Tehran, 11155-9516 Iran

**Keywords:** Computational chemistry, Density functional theory, Reaction mechanisms, Organic chemistry, Theoretical chemistry

## Abstract

In this work, the role of Lewis acid–base (LAB) interaction on the stereoselectivity of the Diels–Alder (DA) reaction has been studied by DFT in gas and solution (dichloromethane) phases. The calculations were performed at the B3LYP/6-311G++ (d, p) level. Two different series of DA reactions were investigated: (1)—three mono-substituted cyclopentadienes + dimethyl(vinyl)borane; (2)—five *α*,*β*-unsaturated carbonyl compounds + cyclopenta-2,4-dien-1-yldimethylborane. The reacting diene and dienophile pairs were chosen to restrict LAB interaction to the exo reaction pathway. It was found that in some of the examined cases, the favorable LAB interaction is so strong that it can lead to a completely exo-selective DA reaction. Furthermore, a novel multistep synthetic method was hypothesized for preparing exo cycloadduct with near 100% stereoselectivity. Our results can open up new avenues toward the rational design of exo-selective DA reactions for synthesizing novel bioorganic compounds.

## Introduction

Since its discovery in 1928, the Diels–Alder (DA) reaction has become a popular method for the synthesis of various cyclic compounds^1–3^. Generally, the stereoselectivity of DA reactions follows the well-known *endo rule*^4^. Recently, there has been a growing number of studies on DA reactions that do not obey the endo rule: reactions exhibiting the unusual exo selectivity^5–10^. Aside from their significant mechanistic implications, exo-selective DA reactions can play a pivotal role in synthesizing molecules with biological activity^11–13^. Exo selectivity can be achieved by manipulating the steric effects^11,14–16^, specific catalysts^17–19^, rigid dienophiles^20–23^, and fluorinated reactants^24,25^.

Despite their pivotal role in organic synthesis, many DA reactions suffer from sluggish kinetics. Consequently, there has been tremendous research on designing and developing efficient catalysts for this reaction in the past 50 years. Among the others, Lewis acids (LAs) are one of the most promising and widely used catalysts. In addition to their remarkable capability to facilitate reaction kinetics, LA catalysts can achieve high regio- and stereoselectivities^18,26^.

In recent decades, the rapid emergence of powerful and high-speed computers has enabled very accurate calculations of molecular energies and structures. Among various computational techniques, density functional theory (DFT) has proven to be a very competent tool for anticipating the kinetics and thermodynamics of reactions. In particular, LA-catalyzed DA reactions have been extensively studied by DFT calculations^27–30^.

In spite of these notable theoretical studies and many others, the rational design of the diene and dienophile for controlling the stereoselectivity of DA reaction has remained vastly unexplored.

Herein, the impact of the Lewis acid–base (LAB) interaction between the diene and dienophile on the stereoselectivity of the DA reaction has been studied. Two different series of DA reactions were investigated: (1)—three mono-substituted cyclopentadienes + dimethyl(vinyl)borane; (2)—five *α*,*β*-unsaturated carbonyl compounds + cyclopenta-2,4-dien-1-yldimethylborane. The reacting diene and dienophile pairs were devised such that the LAB interaction is restricted to the exo reaction pathway. For each of the examined DA reactions, one of the reactants contained a dimethylboryl group (BMe_2_, the LA), and the other carried a Lewis base (LB). These two series of DA reactions have been chosen since they complement each other. In the first series, the BMe_2_ substituent and a lone-pair donor group (LB) are located on the dienophile and diene, respectively, while for the second series, the positions of BMe_2_ and LB are swapped. As a result, studying these series together would make a more thorough examination of the impact of the LAB interaction on the stereoselectivity of DA reactions. Furthermore, similar series of DA reactions have been successfully employed in our previous work to investigate the role of hydrogen bonding on the stereoselectivity of DA reaction.^31^ DFT calculations provided the optimized structures of the transition states (TSs) and final products in the gas and solution phases (dichloromethane). To further explore the kinetics and thermodynamics of the DA reactions, Gibbs free energies of activation and reaction were computed. It was found that the rationally engineered intermolecular LAB interaction can drastically affect the stereoselectivity of the DA reaction leading to almost complete exo selectivity.

## Results and discussion

Two series of DA reactions were devised to explore how the stereoselectivity is affected by the LAB interaction. As noted before, the reactants were chosen to restrict the favorable LAB interaction (between the BMe_2_ group from one molecule and the lone pair donor atom X from the other) to the exo pathway. The optimized structures of the TSs and final products and their associated Gibbs free energies were acquired for each DA reaction. Also, the B…X distances in exo TS and the final product (denoted as *r*(B…X, exo)) were evaluated as a measure of the strength of LAB interaction.

Along the reaction path (prior to the TS) in a bimolecular reaction, the two reactants may form a molecular complex (MC) in which they are held together by weak intermolecular interactions such as van der Waals (vdW) forces, hydrogen bonding, and LAB interaction^32–36^. If the resulting MC is less stable than the corresponding free reactants (that is, the standard Gibbs free energy change associated with the formation of MC from the reactants is + 3 kcal/mol or higher), then its equilibrium concentration would be much lower than the reactants (< 1% at room temperature) and thus the formation of MC can be practically ignored. Consequently, free reactants can be used as the energy reference, as usual. On the other hand, if the MC is more stable than the free reactants (that is, the standard Gibbs free energy change corresponding to the formation of MC from the reactants is − 3 kcal/mol or lower), the equilibrium concentration of the free reactants would be negligible compared to the MC (< 1% at room temperature). Therefore, taking the MC as the energy reference would be necessary*.* In light of the discussion above, the optimized structure of the MC associated with the reacting diene and dienophile was obtained for each DA reaction. Then, based on the relative stability of the reactants and the MC, the energy reference was determined.

### First series of DA reactions

**Figure 1:**
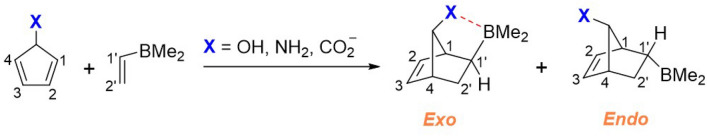
DA reaction of mono-substituted cyclopentadienes and dimethyl(vinyl)borane. The red dashed line represents the LAB interaction.

The first series of DA reactions investigated were between the four mono-substituted cyclopentadienes and dimethyl(vinyl)borane (Fig. 1). The optimized structure of the reactants, MCs, TSs, and final products were obtained for each of the three DA reactions.

Our calculations revealed that the MCs for entries 1-1 to 1-2 were 6.40 and 8.85 kcal/mol higher in Gibbs free energy than the reactants, respectively. However, the Gibbs free energy of 1-3 MC was 5.04 kcal/mol lower than the reactants. Consequently, the energy references for entries 1-1 and 1-2 were the free reactants, whereas for entry 1-3, the corresponding MC was chosen as the energy reference. Figure S1 depicts the optimized structures of the three MCs.

According to the Curtin-Hammet principle, the exo selectivity defined by {[exo]/([exo] + [endo])} × 100 is directly related to the difference between the standard Gibbs free energies of activation of exo and endo stereoisomers (${\Delta}{{\Delta}{\text{G}}}^{\circ \ddag}={\Delta}{\text{G}}_{\text{exo}}^{\circ \ddag}$−${\Delta}{\text{G}}_{\text{endo}}^{{\circ }{\ddag}}$) and can be determined as shown in Eqs. (1) and (2):

1 $$\text{exo selectivity} \, {(\%)} \, \equiv \frac{\left[{\text{exo}}\right]}{\left[{\text{exo}}\right]\text{ + }\left[{\text{endo}}\right]}{ \times }{100 }{=} \, \frac{{\text{k}}_{\text{exo}}}{{\text{k}}_{\text{exo}}+ {\text{k}}_{\text{endo}}} \, {\times}{ 100 }{=} \, \frac{{100}}{{1 + }\frac{{\text{k}}_{\text{endo}}}{{\text{k}}_{\text{exo}}}}$$

2 $$\frac{{\text{k}}_{\text{endo}}}{{\text{k}}_{\text{exo}}}=\frac{{e}^{-\frac{{\Delta}{\text{G}}_{\text{endo}}^{\circ \ddag}}{\text{RT}}}}{{e}^{-\frac{{\Delta}{\text{G}}_{\text{exo}}^{\circ \ddag}}{\text{RT}}}}={e}^{\frac{{\Delta}{\text{G}}_{\text{exo}}^{\circ \ddag}-{\Delta}{\text{G}}_{\text{endo}}^{\circ \ddag}}{\text{RT}}}=\mathrm{exp}\left(\frac{{\Delta}{{\Delta}{\text{G}}}^{\circ \ddag}}{\text{RT}}\right)$$

where ${k}_{exo}$ and ${k}_{endo}$ are the bimolecular rate constants corresponding to exo and endo reaction pathways, respectively (which can be obtained by the Arrhenius equation), *R* is the gas constant, and *T* is the absolute temperature. Table 1 summarizes the results of calculations on TS structures. As can be seen, ${\Delta}{{\Delta}{\text{G}}}^{\circ \ddag}$ values for entries 1-1 to 1-3 are − 0.93, − 4.62, and − 6.61 kcal/mol, respectively. The corresponding exo selectivities at 298.15 K calculated from Eqs. (1) and (2) are 82.7, > 99.9, and > 99.9%, respectively. These results show that the exo selectivity increases in the order of X = OH^−^ < NH_2_ < CO_2_^−^. Interestingly, as shown in Table 1, *r*(B…X, exo) increases in the opposite order. Furthermore, *r*(B…X, exo) for entries 1-2 and 1-3 are shorter than the sum of the van der Waals radii of B and X (3.47 Å for X = N and 3.44 Å for X = O)^37^. These findings imply that the strength of the LAB interaction between B and X in the exo TS is critical in determining the exo selectivity. In particular, the exo selectivities close to 100% obtained for entries 1-2 and 1-3 are presumably related to the strong B…N and B…O interactions in the TS structures, respectively.

**Table 1:** Results of the DFT calculations on the TSs of the three DA reactions outlined in Fig. 1.

Entry	X	${\Delta}{\text{G}}_{\text{exo}}^{\circ \ddag}$ (kcal/mol)	${\Delta}{\text{G}}_{\text{endo}}^{\circ \ddag}$ (kcal/mol)	${\Delta}{{\Delta}{\text{G}}}^{\circ \ddag}$ (kcal/mol)	*r*(B…X, exo) (Å)	Exo selectivity (%)
1-1	OH	33.96	34.89	–0.93	3.07	82.7
1-2	NH_2_	31.35	35.97	–4.62	1.73	> 99.9
1-3	CO_2_^−^	24.48	31.09	–6.61	1.57	> 99.9

Table 2 provides the computation results corresponding to the final products. The difference between the exo and endo standard Gibbs free energies of reaction (${\Delta}{{\Delta}{\text{G}}}^{\circ}\text{ = }{\Delta}{\text{G}}_{\text{exo}}^{\circ}$− ${\Delta}{\text{G}}_{\text{endo}}^{\circ}$) represents the relative thermodynamic stability of exo and endo stereoisomers. It can be observed that ${\Delta}{{\Delta}{\text{G}}}^{\circ}$ values for entries 1-1 to 1-3 are − 2.31, − 8.02, and − 26.20 kcal/mol, respectively. Thus, the relative stability of the exo stereoisomer increases in the order of X = OH^−^ < NH_2_ < CO_2_^−^. However, the increase of *r*(B…X, exo) shows an opposite trend. Moreover, *r*(B…X, exo) for entries 1-2 and 1-3 are shorter than the sum of the van der Waals radii of B and X. As one can see, these trends are very similar to those observed for TSs. Hence, it can be inferred that ${\Delta}{{\Delta}{\text{G}}}^{\circ}$ is primarily affected by the strength of the LAB interaction of B and X. Especially the fairly large absolute values of ${\Delta}{{\Delta}{\text{G}}}^{\circ}$ (> 8 kcal/mol) for entries 1-2 and 1-3 are presumably the results of strong B…N and B…O interactions in the final products, respectively.

**Table 2:** Results of the DFT calculations on the final products of the three DA reactions outlined in Fig. 1.

Entry	X	${{\Delta}{\text{G}}}_{exo}^{^\circ }$ (kcal/mol)	${{\Delta}{\text{G}}}_{endo}^{^\circ }$ (kcal/mol)	${\Delta}{{\Delta}{\text{G}}}^{\circ}$ (kcal/mol)	*r*(B…X, exo) (Å)
1-1	OH	0.14	2.45	− 2.31	2.95
1-2	NH_2_	− 1.57	6.45	− 8.02	1.75
1-3	CO_2_^−^	− 10.67	15.53	− 26.20	1.58

**Figure 2:**
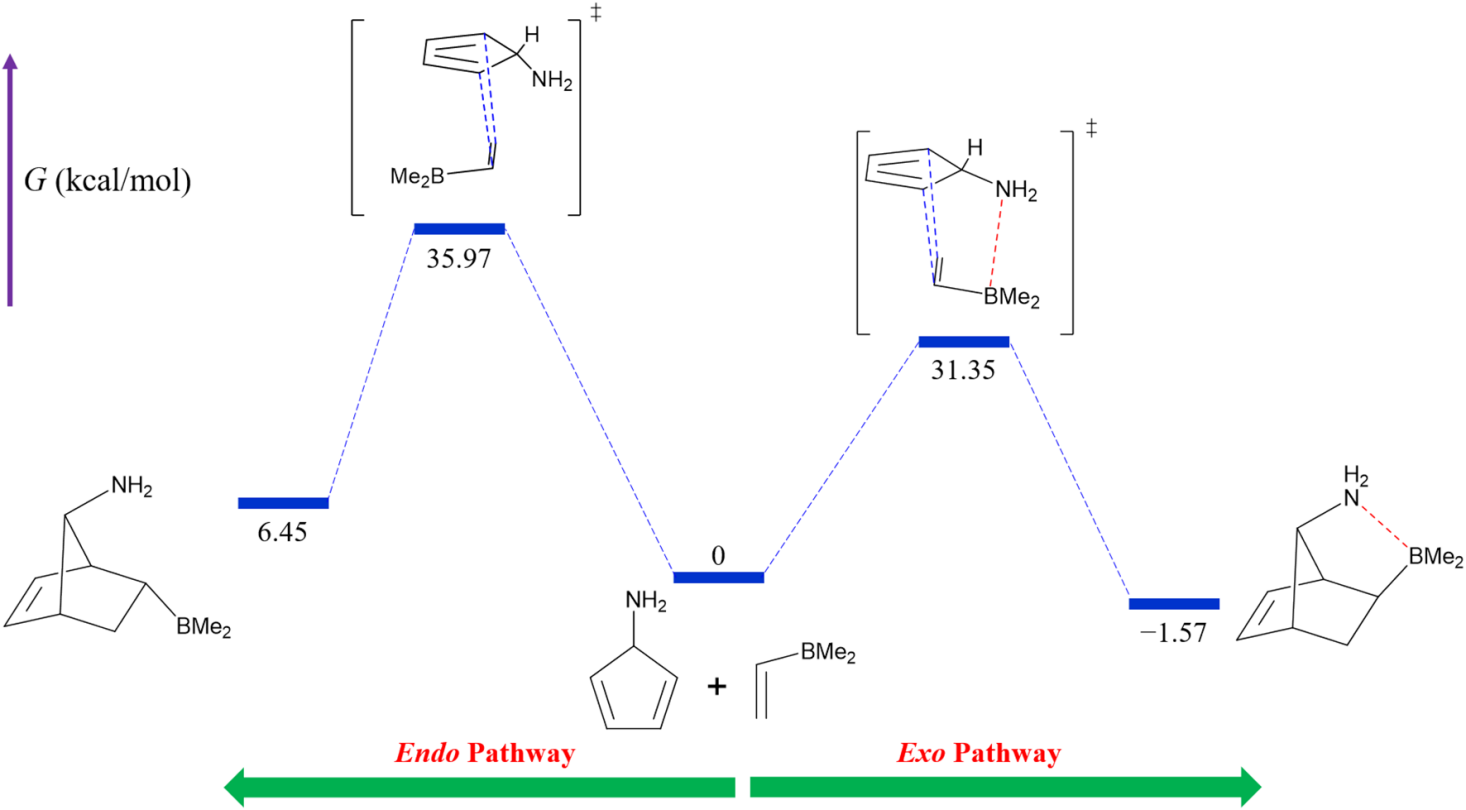
The reaction energy diagram for entry 1-2. Numbers represent the Gibbs free energies in kcal/mol relative to the reactants. The blue and red dashed lines represent the newly forming C–C bonds and the LAB interaction, respectively.

**Figure 3:**
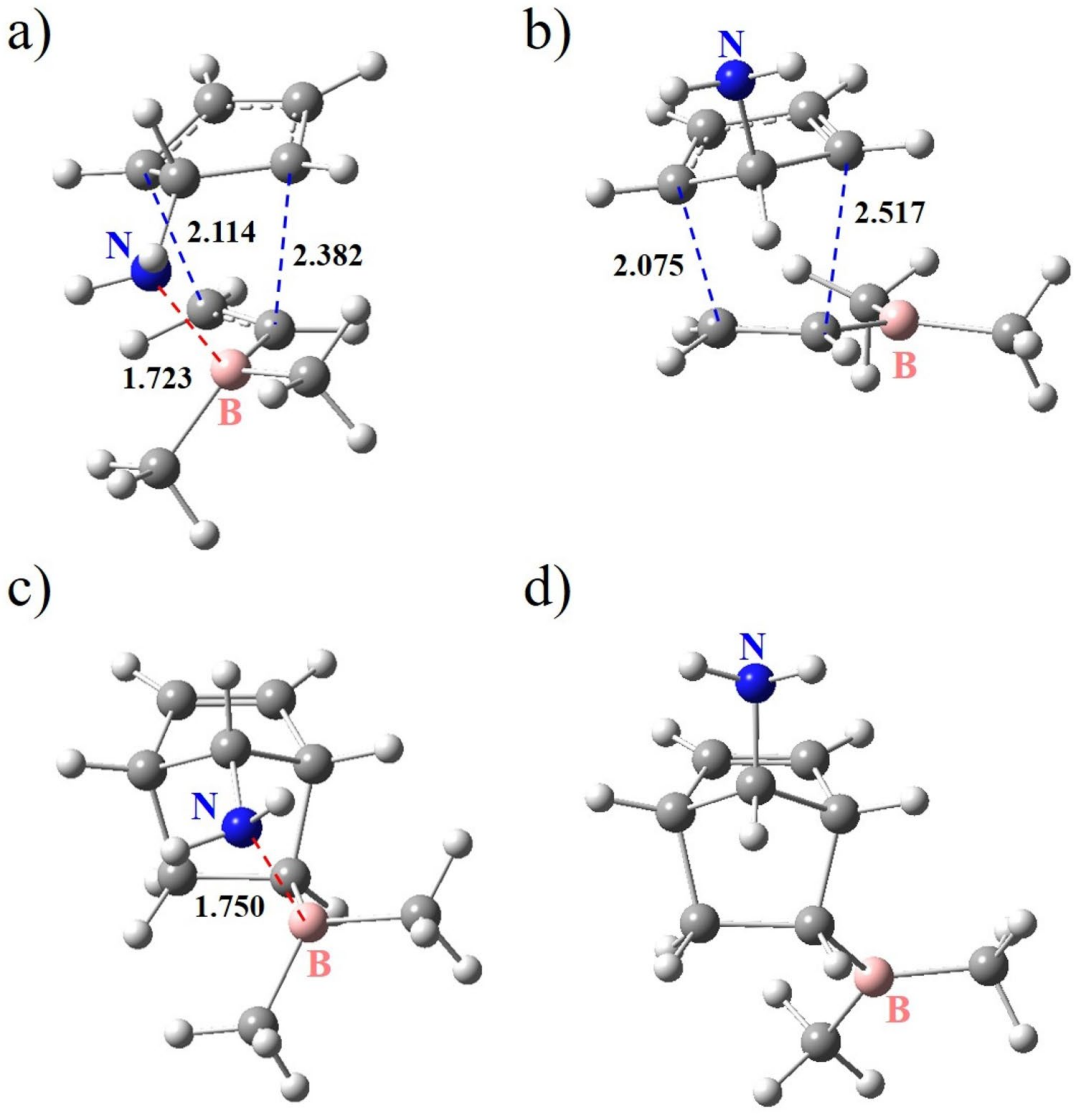
The optimized structures of the exo and endo TSs (**a** and **b**) and the corresponding final products (**c** and **d**) obtained for entry 1-2. The blue and red dashed lines represent the newly forming C–C bonds and the LAB interaction, respectively. The numbers indicate the adjacent bond length or atom–atom distance in angstroms.

The reaction energy diagram for entry 1-2 is shown in Fig. 2 as a representative example. Figure 3 depicts the optimized structures of the associated TSs and final products.

These findings indicate that the exo stereoisomer is the kinetically and thermodynamically dominant product for entries 1-2 and 1-3 due to the strong LAB interaction.

### Second series of DA reactions

**Figure 4:**
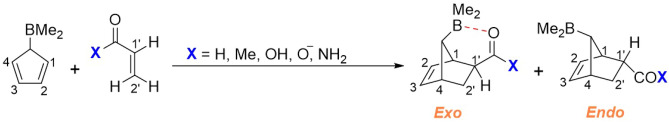
DA reaction of cyclopenta-2,4-dien-1-yldimethylborane and *α*,*β*-unsaturated carbonyl compounds. The red dashed line represents the LAB interaction.

The second series of DA reactions studied were between five *α*,*β*-unsaturated carbonyl compounds and cyclopenta-2,4-dien-1-yldimethylborane (Fig. 4).

MCs for entries 2-1 to 2-3 were calculated to be 23.40, 5.93, and 8.83 kcal/mol lower in Gibbs free energy than the reactants, respectively. On the other hand, for entries 2-4 and 2-5, MCs were 15.07 and 6.63 kcal/mol higher in Gibbs free energy than the corresponding reactants, respectively. Accordingly, the energy references for entries 2-1 to 2-3 were the reactants, while MCs were taken as the energy references for entries 2-4 and 2-5. Figure S2 shows the optimized structures of the five MCs.

The results of calculations on TSs are given in Table 3. As can be seen, ${\Delta}{{\Delta}{\text{G}}}^{\circ \ddag}$ for entries 2-1 to 2-5 are − 10.81, − 11.26, − 0.86, − 26.30, and − 9.09, respectively. The corresponding exo selectivities at 298.15 K are > 99.9, > 99.9, 81.1, > 99.9, and > 99.9%, respectively. Consequently, the exo selectivity increases in the order of X = OH < NH_2_ < H < Me < O^−^. As shown in Table 3, *r*(B…O, exo) values exhibit an opposite trend. As previously discussed, these observations suggest that the strength of LAB interaction between B (from the diene) and O of the carbonyl is the primary factor in determining the exo selectivity. Furthermore, the much higher ${\Delta}{{\Delta}{\text{G}}}^{\circ \ddag}$ absolute values compared to the first series of DA reactions (entries 1-1 to 1-3) imply stronger LAB interactions.

**Table 3:** Results of the DFT calculations on the TSs of the five DA reactions outlined in Fig. 4.

Entry	X	${\Delta}{\text{G}}_{\text{exo}}^{{\circ}{\ddag}}$ (kcal/mol)	${\Delta}{\text{G}}_{\text{endo}}^{{\circ}{\ddag}}$ (kcal/mol)	${\Delta}{{\Delta}{\text{G}}}^{\circ \ddag}$ (kcal/mol)	r(B…O, exo) (Å)	exo selectivity (%)
2-1	H	27.58	38.39	−10.81	1.603	> 99.9
2-2	Me	26.92	38.18	−11.26	1.596	> 99.9
2-3	OH	35.30	36.16	−0.86	1.624	81.1
2-4	O^−^	29.27	55.57	−26.30	1.559	> 99.9
2-5	NH_2_	22.71	31.80	−9.09	1.615	> 99.9

Table 4 shows the calculation results corresponding to the final products, in which ${\Delta}{{\Delta}{\text{G}}}^{\circ}$ values for entries 2-1 to 2-5 are 1.33, − 5.04, 0.97, − 25.74, and − 2.94 kcal/mol, respectively. Thus, the absolute value of ${\Delta}{{\Delta}{\text{G}}}^{\circ}$ increases in the order of X = H < OH^−^ < NH_2_ < Me < O^−^. According to Table 4, this order is almost reversed for *r*(B…O, exo) values. These two trends imply that the ${\Delta}{{\Delta}{\text{G}}}^{\circ}$ is primarily determined by the strength of the LAB interaction between B of the diene and O of the carbonyl.

**Table 4:** Results of the DFT calculations on the final products of the five DA reactions outlined in Fig. 4.

Entry	X	${{\Delta}{\text{G}}}_{exo}^{^\circ }$ (kcal/mol)	${{\Delta}{\text{G}}}_{endo}^{^\circ }$ (kcal/mol)	${\Delta}{{\Delta}{\text{G}}}^{\circ}$ (kcal/mol)	r(B…O, exo) (Å)
2-1	H	9.15	7.82	1.33	1.773
2-2	Me	8.16	13.20	− 5.04	1.741
2-3	OH	9.40	8.43	0.97	1.874
2-4	O^−^	− 2.16	23.58	− 25.74	1.581
2-5	NH_2_	− 1.32	1.62	− 2.94	1.731

**Figure 5:**
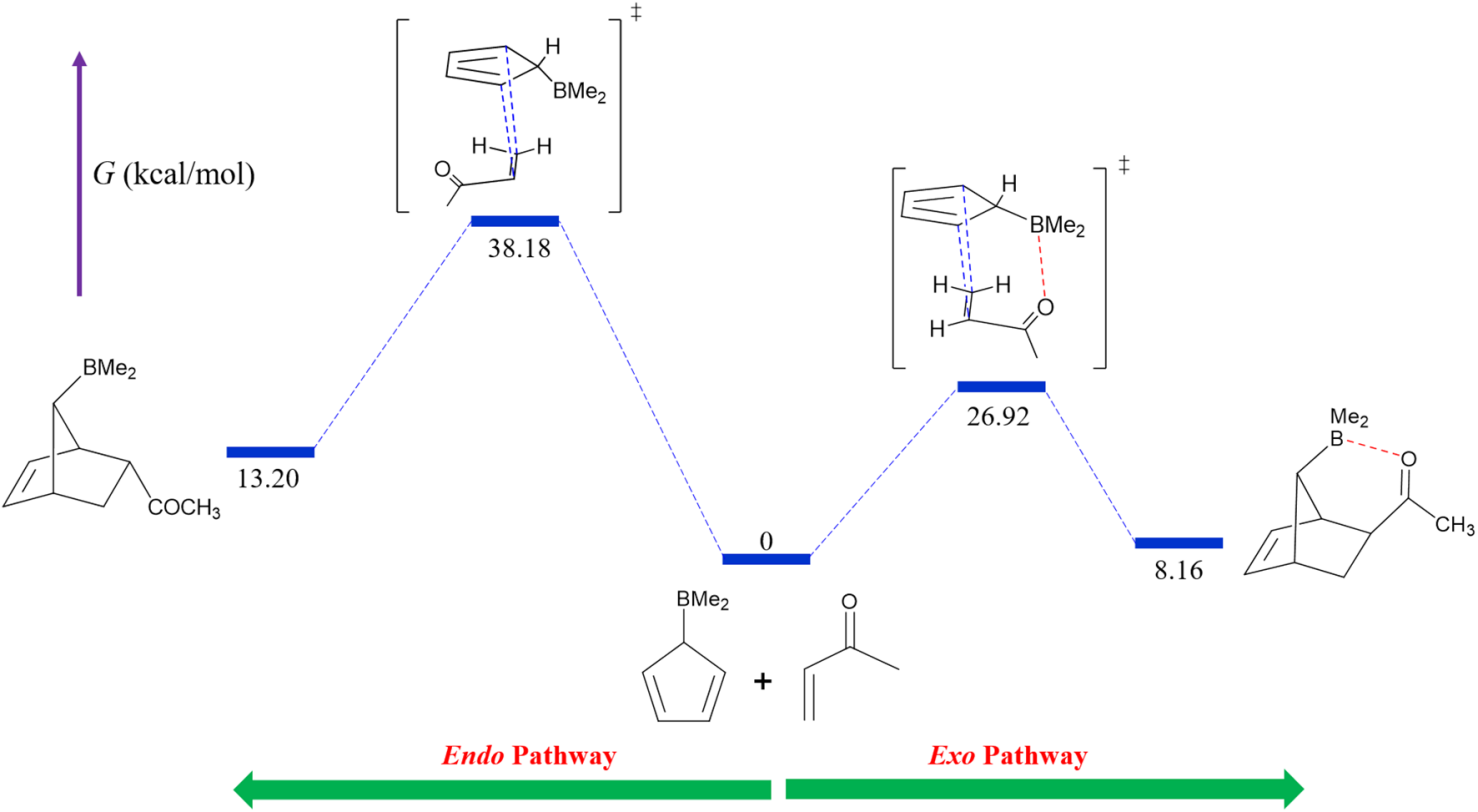
The reaction energy diagram for entry 2-2. Numbers represent the Gibbs free energies in kcal/mol relative to the reactants. The blue and red dashed lines represent the newly forming C–C bonds and the LAB interaction, respectively.

**Figure 6:**
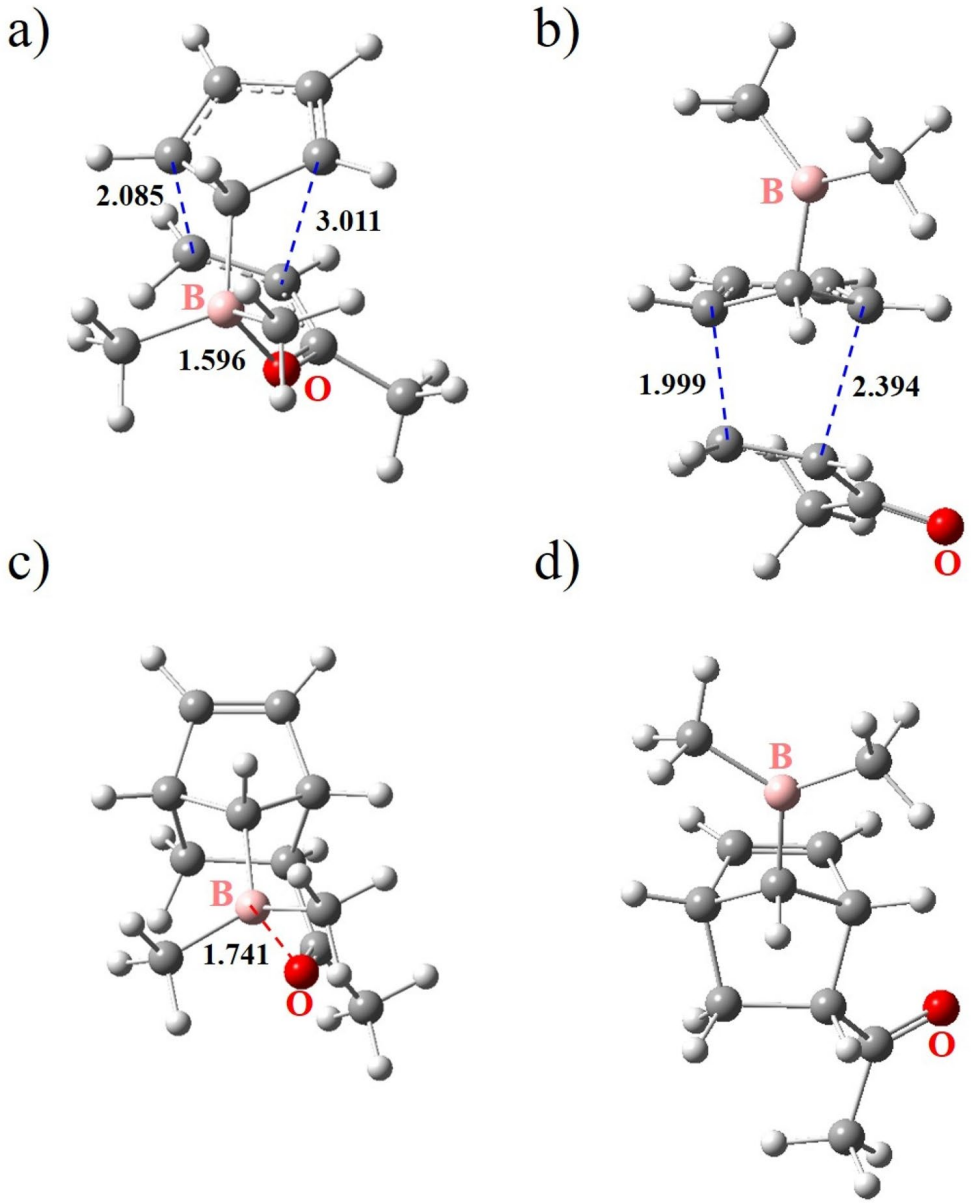
The optimized structures of the exo and endo TSs (**a** and **b**) and the corresponding final products (**c** and **d**) obtained for entry 2-2. The blue and red dashed lines represent the newly forming C–C bonds and the LAB interaction, respectively. The numbers indicate the adjacent bond length or atom–atom distance in angstroms.

The reaction energy diagram for entry 2-2 is given in Fig. 5 as a representative example. Figure 6 illustrates the optimized structures of the associated TSs and final products.

Overall, the data in Tables 3 and 4 clearly demonstrate that the exo stereoisomer is the dominant kinetic and thermodynamic product for entries 2-2, 2-4, and 2-5 due to the strong LAB interaction.

### Solution-phase calculations

To complement the gas phase calculations discussed in previous sections, solution-phase calculations in dichloromethane (DCM) were performed on MCs, reactants, TSs, and final products of the two series of DA reactions. It should be noted that the optimized structures in DCM were assumed to be the same as those obtained in the gas phase since the energy differences were negligible. Our calculations indicated that MCs for entries 1-1 to 1-3 were 8.88, 7.66, and 6.92 kcal/mol higher in Gibbs free energy than the reactants, respectively. For entries 2-1 to 2-4, MCs were 28.83, 22.67, 11.36, and 2.14 kcal/mol higher in Gibbs free energy than the reactants, respectively. However, for entry 2-5, MC was 4.95 kcal/mol lower in Gibbs free energy than the reactants. Consequently, the reactants were chosen as the energy reference for entries 1-1 to 1-3 and 2-1 to 2-4, whereas for entry 2-5, MC was chosen as the energy reference. Tables 5 and 6 summarize the results of calculations on TSs of the first and second series of DA reactions, respectively. Notably, the exo selectivity shows the same trend as in gas-phase calculations for both series of DA reactions. However, ${\Delta}{{\Delta}{\text{G}}}^{\circ \ddag}$ absolute values in the solution are smaller than those in the gas phase (except for entries 1-2, 2-3, and 2-5).

**Table 5:** Results of the solution-phase DFT calculations on the TSs of the three DA reactions outlined in Fig. 1.

Entry	X	${\Delta}{\text{G}}_{\text{exo}}^{{\circ}{\ddag}}$ (kcal/mol)	${\Delta}{\text{G}}_{\text{endo}}^{{\circ}{\ddag}}$ (kcal/mol)	${\Delta}{{\Delta}{\text{G}}}^{\circ \ddag}$ (kcal/mol)	exo selectivity (%)
1-1	OH	35.69	35.72	− 0.03	52.4
1-2	NH_2_	31.70	37.03	− 5.33	> 99.9
1-3	CO_2_^−^	31.76	36.08	− 4.32	> 99.9

**Table 6:** Results of the solution-phase DFT calculations on the TSs of the five DA reactions outlined in Fig. 4.

Entry	X	${\Delta}{\text{G}}_{\text{exo}}^{{\circ}{\ddag}}$ (kcal/mol)	${\Delta}{\text{G}}_{\text{endo}}^{{\circ}{\ddag}}$ (kcal/mol)	${\Delta}{{\Delta}{\text{G}}}^{\circ \ddag}$ (kcal/mol)	Exo selectivity (%)
2-1	H	29.14	37.08	− 7.94	> 99.9
2-2	Me	28.69	37.94	− 9.25	> 99.9
2-3	OH	34.72	36.46	− 1.74	95.0
2-4	O^−^	28.38	44.66	− 16.28	> 99.9
2-5	NH_2_	21.43	28.89	− 7.46	> 99.9

The results of calculations on the final products of the first and second series of DA reactions are shown in Tables 7 and 8, respectively. Again, the observed trend in ${\Delta}{{\Delta}{\text{G}}}^{\circ}$ is similar to that of the gas phase for both series of DA reactions. Moreover, ${\Delta}{{\Delta}{\text{G}}}^{\circ}$ values in solution are roughly similar to those in the gas phase (except for entries 1-3 and 2-4).

**Table 7:** Results of the solution-phase DFT calculations on the final products of the three DA reactions outlined in Fig. 1.

Entry	X	${{\Delta}{\text{G}}}_{exo}^{^\circ }$ (kcal/mol)	${{\Delta}{\text{G}}}_{endo}^{^\circ }$ (kcal/mol)	${\Delta}{{\Delta}{\text{G}}}^{\circ}$ (kcal/mol)
1-1	OH	2.37	5.00	− 2.63
1-2	NH_2_	− 0.97	7.68	− 8.65
1-3	CO_2_^−^	− 3.78	10.59	− 14.37

**Table 8:** Results of the solution-phase DFT calculations on the final products of the five DA reactions outlined in Fig. 4.

Entry	X	${{\Delta}{\text{G}}}_{exo}^{^\circ }$ (kcal/mol)	${{\Delta}{\text{G}}}_{endo}^{^\circ }$ (kcal/mol)	${\Delta}{{\Delta}{\text{G}}}^{\circ}$ (kcal/mol)
2-1	H	13.38	11.35	2.03
2-2	Me	9.54	14.05	− 4.51
2-3	OH	10.81	9.58	1.23
2-4	O^−^	− 2.13	13.16	− 15.29
2-5	NH_2_	− 3.76	0.82	− 4.58

Overall, solvent calculations indicated that the trend in gas-phase exo selectivities is mainly preserved in DCM, even though the gas-phase ${\Delta}{{\Delta}{\text{G}}}^{{\circ}{\ddag}}$ and ${\Delta}{{\Delta}{\text{G}}}^{\circ}$ absolute values were smaller than those in DCM.

### A novel synthetic strategy for preparing exo cycloadducts

**Figure 7:**
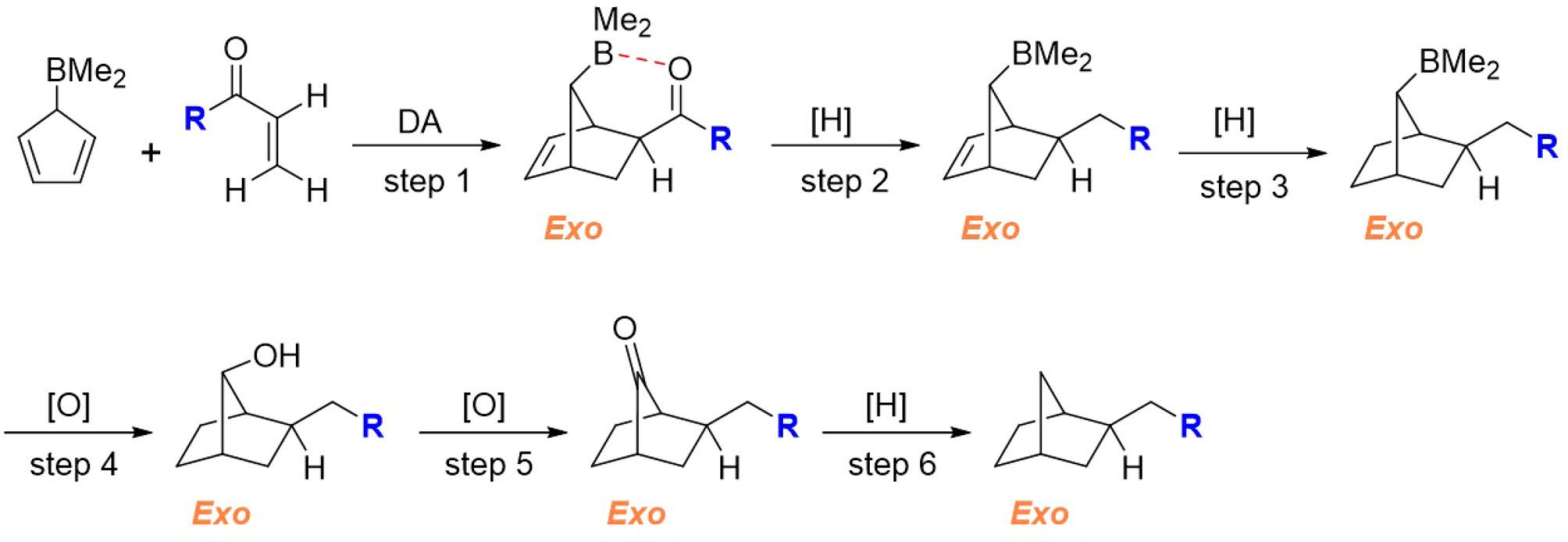
A proposed synthetic route for the stereoselective synthesis of exo-2-alkylbicyclo[2.2.1]heptanes. The red dashed line indicates the LAB interaction.

Based on the findings of this study, a synthetic strategy has been proposed for preparing stereochemically-pure exo cycloadducts. As depicted in Fig. 7, the first step is the DA reaction of an *α*,*β*-unsaturated carbonyl compound and cyclopenta-2,4-dien-1-yldimethylborane, which yields almost exclusively the exo stereoisomer (according to our calculations). The next step involves the reduction of carbonyl to CH_2_, which can be performed with high yield and under mild conditions with a variety of methods such as Et_3_SiH + catalytic amounts of B(C_6_F_5_)_3_^38^, polymethylhydrosiloxane** + **catalytic amounts of B(C_6_F_5_)_3_,^39^ and ruthenium (II)-catalyzed Wolf-Kishner reaction^40^. In the third step, the C=C bond is transformed into a C–C bond by catalytic hydrogenation (e.g., H_2_ over Pt or Pd/C catalyst). Finally, the BMe_2_ group is replaced by H in three consecutive steps: (1)—Oxidation of BMe_2_ to OH by H_2_O_2_/OH^−^ reagent (step 4)^41,42^. This is the second step of the well-known hydroboration-oxidation reaction, which transforms an alkene into alcohol. (2)—Oxidation of the obtained alcohol to a ketone using common reagents under mild conditions, such as oxalyl chloride (Swern oxidation), pyridinium chlorochromate (PCC, Corey-Suggs reagent), or Dess-Martin periodinane (DMP) (step 5). (3)—Reduction of the ketone to CH_2_ by the mild and efficient methods described above (step 6).

## Conclusions

A series of dienes and dienophiles were designed to theoretically examine the role of intermolecular LAB interaction on the stereoselectivity of DA reaction. DFT calculations revealed that the favorable interaction between the BMe_2_ group (LA) of one reactant and the lone-pair donor atom (LB) of the other reactant could significantly affect the stereoselectivity of the DA reaction. Remarkably, when the LAB interaction is strong enough, it can lead to a completely exo-selective DA reaction in the gas and solution phases. Inspired by these results, a novel method was proposed to synthesize stereochemically pure exo-2-alkylbicyclo[2.2.1]heptanes. Our findings can provide new insights into the rational design of exo-selective DA reactions.

## Methods

Density functional theory (DFT) was employed to calculate the molecular structures and energies. First, the conformers with the lowest energy were found at the relative energy of 0–10 kcal/mol range for the reaction components using the Merck Molecular Force field (MMFF) in Spartan 14 software^43^. Then, the most stable conformers were optimized by the DFT calculations at the B3LYP/6-311++ G (d, p) computational level^44–46^, in which “p” and “d” denote polarized and diffuse functions, respectively. The diffuse function is vital for molecules with lone pairs and anions^47–49^. We have justified the use of MMFF and B3LYP computational levels in our previous works^50,51^. Since the energy ordering might vary between MMFF and DFT, DFT optimization was performed on the lowest energy MMFF conformers at the relative energy range of 1–3 kcal/mol. The lack of imaginary frequencies verified the existence of the local energy minima of the structures under consideration. The solution-phase calculations were performed using the conductor-like polarizable continuum model (CPCM)^52,53^. All calculations were carried out at 1.00 atm and 298.15 K.

## Supplementary Information

Supplementary Information.

## Data Availability

All data generated or analyzed during this study are included in this published article [and its supplementary information files].
