# Supplementary material for: Inducing high exo selectivity in Diels–Alder reaction by dimethylborane substituent: a DFT study
Source: Sci Rep. 2022 Dec 23;12:22225. doi: 10.1038/s41598-022-26685-y (PMC9789069; doi:10.1038/s41598-022-26685-y)
Supplement: Supplementary file 1 — Supplementary Information. [file 41598_2022_26685_MOESM1_ESM.docx]

**Inducing High Exo Selectivity in Diels-Alder Reaction by Dimethylborane Substituent: A DFT Study**

*Davood Taherinia*^1^**, Alireza Fattahi* ^1^***

^1^ Department of Chemistry, Sharif University of Technology, Tehran 11155-9516, Iran

*email: taherinia@sharif.edu

*email: fattahi@sharif.edu

**Table of Contents**

[**Figure S1. Optimized Structures of MCs for Entries 1-1 to 1-3** 2](#_Toc121046467)

[**Figure S2. Optimized Structures of MCs for Entries 2-1 to 2-5** 3](#_Toc121046468)

[**Cartesian Coordinates, Absolute Internal Energies (*E*), and Absolute Gibbs Free Energies (*G*) of Products at 298.15 K** 4](#_Toc121046469)

[**Cartesian Coordinates, Imaginary Frequencies, Absolute Internal Energies (*E*), and Absolute Gibbs Free Energies (*G*) of TS Structures at 298.15 K** 16](#_Toc121046470)

[**Cartesian Coordinates, Absolute Internal Energies (*E*), and Absolute Gibbs Free Energies (*G*) of MCs at 298.15 K** 29](#_Toc121046471)


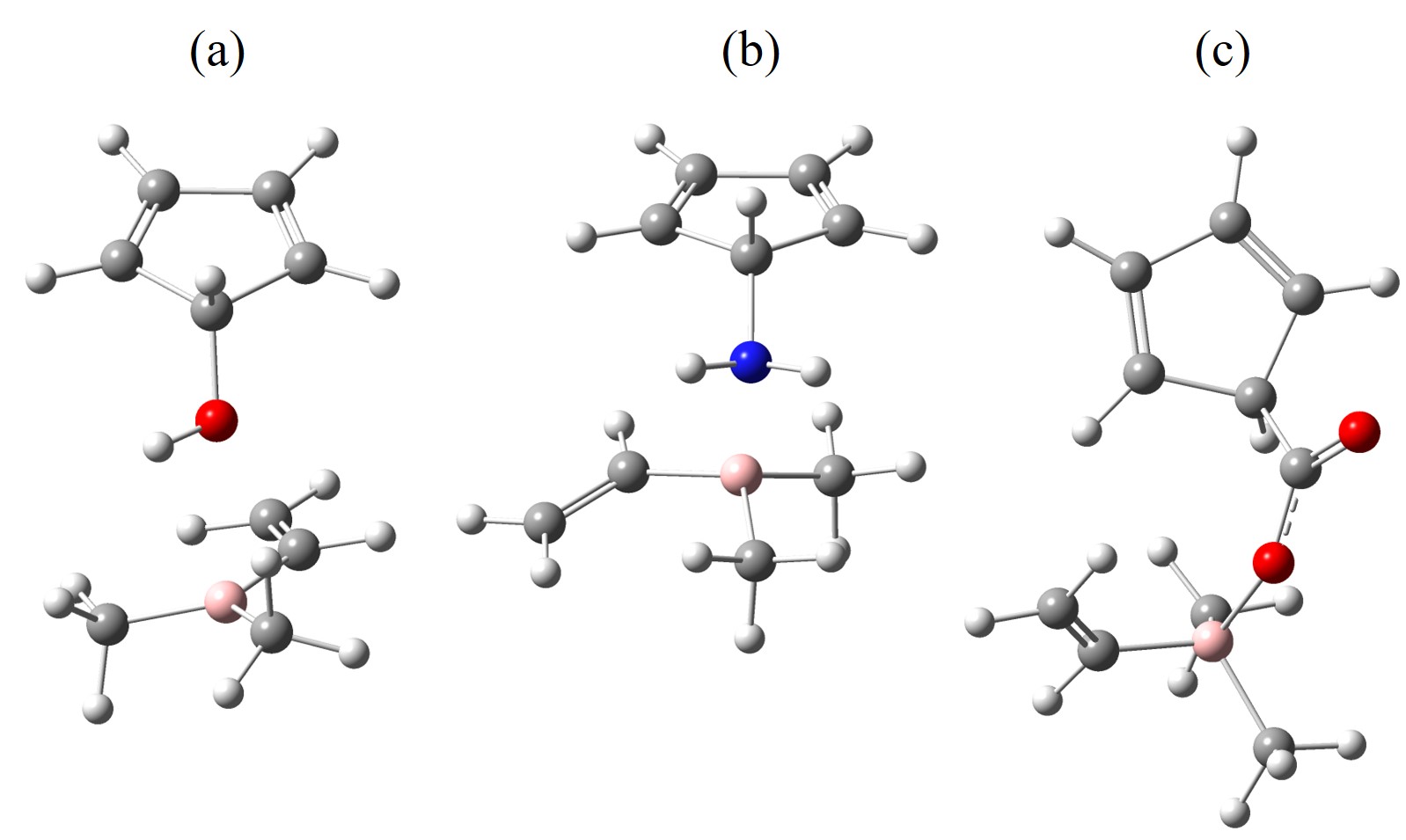


**Figure S1.** The optimized structures of the MCs for: (a) entry 1-1; (b) entry 1-2; and (c) entry 1-3.


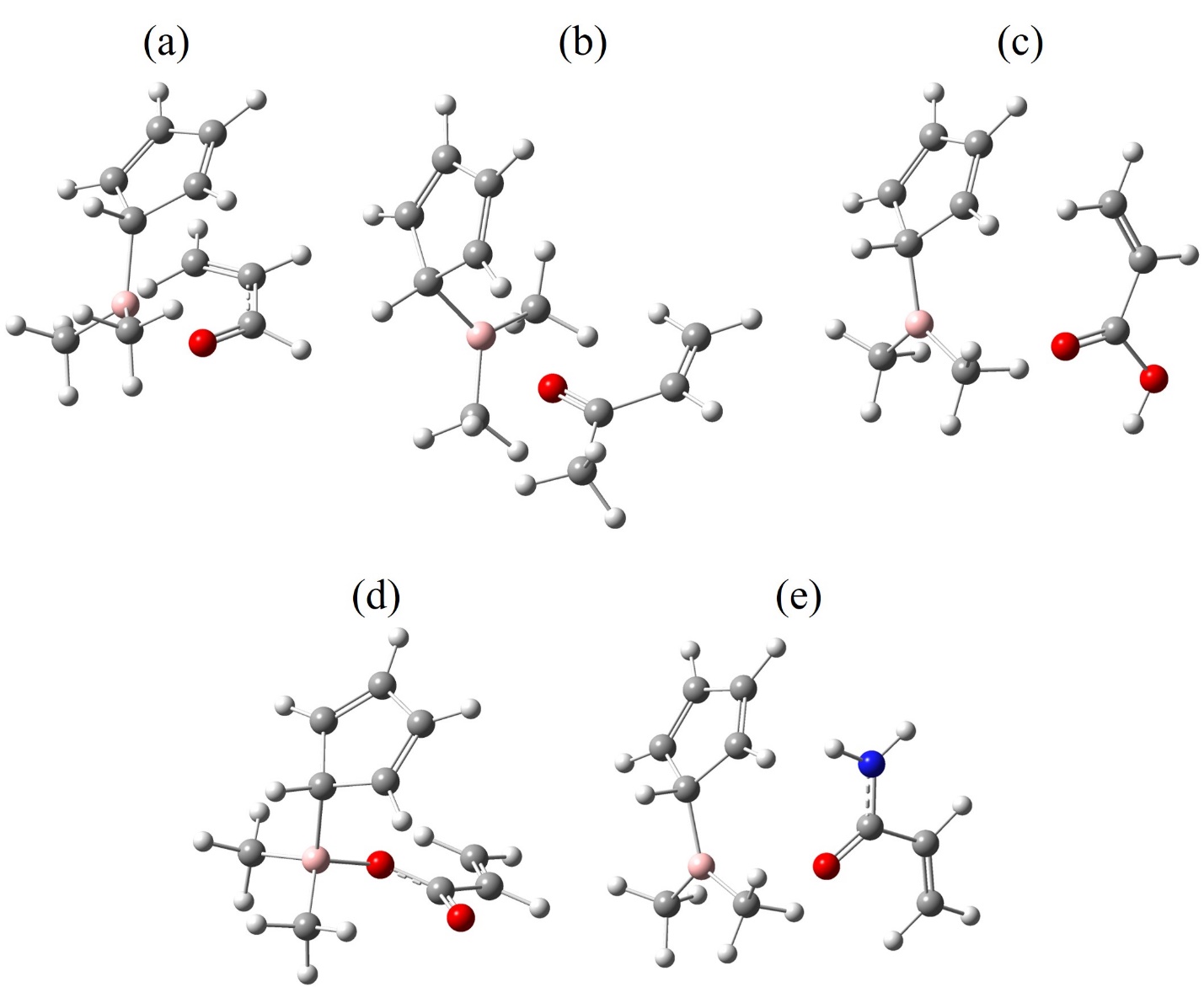


**Figure S2.** The optimized structures of the MCs for: (a) entry 2-1; (b) entry 2-2; (c) entry 2-3; (d) entry 2-4; and (e) entry 2-5.

# **Cartesian Coordinates, Absolute Internal Energies (*E*), and Absolute Gibbs Free Energies (*G*) of Products at 298.15 K**

Name: 1-1 endo

Absolute *E* (Hartree): −451.924332

Absolute *G* (Hartree): −451.974899

Coordinates:

X Y Z

C 1.17483900 -0.79099100 1.35539100

C 1.48602200 -1.10454600 -0.10084300

C 0.48289800 0.91705500 -0.03219600

C 0.58082500 0.40553100 1.39727200

C 1.77232100 0.30745500 -0.64553600

C 0.10910500 -1.38070100 -0.79201700

H 0.25694500 -1.72982700 -1.81807500

H -0.44577900 -2.15517300 -0.26075000

C -0.59952500 0.01982900 -0.76196400

H 2.25304400 -1.85622800 -0.28366100

H 0.33797900 1.99019500 -0.15888100

H 0.16262500 0.90003900 2.26520400

H 1.33409300 -1.47376000 2.18038700

O 3.01123100 0.81049800 -0.17404700

H 3.10606600 1.72194500 -0.46711600

H 1.74856800 0.33788800 -1.74294200

B -2.06997300 0.15853500 -0.19559600

C -2.70621500 1.59087200 -0.01694400

H -2.94027800 1.75196900 1.04508800

H -2.09474100 2.43159200 -0.35437000

H -3.67651700 1.64233700 -0.52750500

C -2.92736800 -1.10970800 0.17185400

H -2.47114300 -1.60070500 1.04377100

H -3.97157500 -0.89932700 0.41823500

H -2.90265200 -1.86514400 -0.62311500

H -0.67402600 0.39877200 -1.79601800

Name: 1-1 exo

Absolute *E* (Hartree): −451.931552

Absolute *G* (Hartree): −451.978586

Coordinates:

X Y Z

C 2.56565000 0.14226000 -0.63046000

C 1.67270900 0.89829900 0.33235000

C 0.63233500 -1.04193500 -0.18041100

C 1.94449400 -1.00441200 -0.93891200

C 1.07097800 -0.31024200 1.10397200

C 0.41926000 1.37786500 -0.46904800

H -0.19366200 2.02779900 0.15792100

H 0.71131500 1.94560100 -1.35455100

C -0.32736400 0.04228700 -0.80826400

H -0.34533200 -0.10778400 -1.90030600

H 2.15350600 1.67480400 0.93017500

H 0.18685600 -2.02510200 -0.04231500

H 2.26410700 -1.74917000 -1.65756200

H 3.49256700 0.51851500 -1.04546100

B -1.85615300 -0.05366800 -0.39890300

C -2.50411100 -1.45592700 -0.07808000

H -2.25079400 -1.70113500 0.96356900

H -3.59470700 -1.47327100 -0.15636300

H -2.09768700 -2.27474000 -0.68198300

C -2.75998700 1.23850900 -0.35672900

H -2.36637900 2.09931200 -0.90429500

H -3.78803500 1.05015600 -0.68196400

H -2.83199500 1.54623800 0.69786300

O -0.05261800 -0.02138200 1.93122100

H 0.24737300 0.45505200 2.71172100

H 1.83079300 -0.86710600 1.66178900

Name: 1-2 endo

Absolute *E* (Hartree): −432.036564

Absolute *G* (Hartree): −432.084048

Coordinates:

X Y Z

C 1.13877800 -0.76768300 1.38919700

C 1.46238800 -1.12352200 -0.05430200

C 0.49084700 0.90727000 -0.05781000

C 0.56452000 0.43805300 1.38806900

C 1.77882800 0.27311600 -0.64608400

C 0.08896500 -1.40468000 -0.75128500

H 0.24379400 -1.77666400 -1.76845400

H -0.47762900 -2.16368500 -0.20954100

C -0.60450900 0.00283600 -0.75764600

H 2.21221600 -1.90241600 -0.20383500

H 0.35789900 1.97776900 -0.21417000

H 0.14459900 0.96628000 2.23503000

H 1.27744000 -1.42880700 2.23544800

H 1.71719000 0.24477300 -1.74492900

B -2.07601200 0.16846900 -0.20421000

C -2.68803900 1.61082100 -0.01873100

H -2.95065000 1.75982200 1.03821300

H -2.05158200 2.44461400 -0.32503700

H -3.64177400 1.68844900 -0.55688400

C -2.96739500 -1.08406800 0.13802500

H -2.55046800 -1.56765500 1.03373600

H -4.01628100 -0.85547200 0.34582900

H -2.92446000 -1.85101500 -0.64453700

H -0.67942500 0.35196700 -1.80281500

N 3.02259000 0.86501900 -0.16715600

H 3.82161800 0.30720400 -0.45063200

H 3.15313700 1.79449400 -0.55287400

Name: 1-2 exo

Absolute *E* (Hartree): −432.052410

Absolute *G* (Hartree): −432.096825

Coordinates:

X Y Z

C -2.68599200 -0.16729000 -0.04485700

C -1.49785900 -0.91632900 0.53198000

C -0.74581500 1.07331400 -0.22737400

C -2.22285300 0.98950200 -0.53563700

C -0.69401400 0.28044500 1.10168500

C -0.62494900 -1.30836400 -0.71943400

H 0.05759400 -2.12927400 -0.49438800

H -1.27446600 -1.64191700 -1.53179000

C 0.13368800 0.02079100 -1.01805900

H 0.21368600 0.24518200 -2.08315900

H -1.72484400 -1.73039300 1.22276100

H -0.32666700 2.07684100 -0.21369900

H -2.77462800 1.71936400 -1.11476400

H -3.67804000 -0.58574800 -0.15903100

B 1.54670300 0.03721800 -0.17851500

C 2.37067500 1.43592400 -0.19222200

H 3.19402300 1.43557200 0.53616400

H 2.84176000 1.57458900 -1.17337400

H 1.78205600 2.34241100 -0.00591400

C 2.54540000 -1.23108600 -0.30090000

H 3.00450000 -1.24841700 -1.29733600

H 3.37945100 -1.16569700 0.41119800

H 2.08549700 -2.21754200 -0.16125800

H -1.17143700 0.75634000 1.95997700

N 0.74185100 -0.04844400 1.37296100

H 1.15349900 0.62628000 2.01302300

H 0.84186700 -0.96602100 1.80234700

Name: 1-3 endo

Absolute *E* (Hartree): −564.759896

Absolute *G* (Hartree): −564.813661

Coordinates:

X Y Z

C 0.50695200 -1.24400600 1.17022900

C 0.81030400 -1.36205500 -0.31445300

C 0.03431200 0.72140400 0.05910800

C 0.04407500 -0.00797500 1.39158800

C 1.27369000 0.08418800 -0.61622600

C -0.58040400 -1.38620700 -1.04312000

H -0.44367400 -1.59286400 -2.11046100

H -1.23548600 -2.16626900 -0.64306000

C -1.13911400 0.05971900 -0.82778600

H 1.50045400 -2.15371500 -0.59883700

H 0.01502800 1.80750000 0.10187800

H -0.31494000 0.39991600 2.32990900

H 0.59725900 -2.04896200 1.89087800

H 1.27830600 0.28252900 -1.69675400

B -2.54175600 0.30354600 -0.17274100

C -3.16864300 1.75879900 -0.19998200

H -3.32649600 2.11715800 0.82697900

H -2.57398800 2.50724100 -0.73119800

H -4.17150700 1.72974100 -0.65011300

C -3.36365200 -0.85060600 0.53021400

H -2.72364900 -1.42158600 1.21257600

H -4.25262900 -0.51411200 1.07480200

H -3.69263500 -1.57281600 -0.23119500

H -1.10781200 0.60905100 -1.77929500

C 2.67476300 0.51682900 -0.07138600

O 3.56497500 -0.36410300 -0.13351400

O 2.76088100 1.70896900 0.30832300

Name: 1-3 exo

Absolute *E* (Hartree): −564.807344

Absolute *G* (Hartree): −564.855422

Coordinates:

X Y Z

C -2.79273400 -0.61393100 0.18713400

C -1.76115400 0.29410400 0.83426900

C -0.81818100 -0.58501300 -1.01398200

C -2.21816100 -1.15338800 -0.89740400

C -1.03182900 0.83494700 -0.43400100

C -0.66685000 -0.66815000 1.41455700

H -0.00114400 -0.11967300 2.08505100

H -1.12663900 -1.47526500 1.99470000

C 0.08911100 -1.16362900 0.13656200

H 0.12916400 -2.25822600 0.09033100

H -2.13851400 1.04432800 1.53094000

H -0.36702800 -0.64252000 -2.00354100

H -2.62181500 -1.94408100 -1.52188800

H -3.74510300 -0.88419800 0.63118600

B 1.58922200 -0.50159700 -0.00230600

C 2.34917100 -0.88815900 -1.40117100

H 3.32908700 -0.39495200 -1.46293800

H 2.53315400 -1.97099000 -1.46920800

H 1.79753200 -0.59985300 -2.30627200

C 2.56385200 -0.82767300 1.26947200

H 2.79445700 -1.90207200 1.32612000

H 3.52288300 -0.29971500 1.17415400

H 2.14030300 -0.53859700 2.24073400

H -1.70675200 1.44916200 -1.03368800

C 0.22733200 1.67121200 -0.16207300

O 1.36930600 1.06095300 -0.00247600

O 0.11456300 2.89438700 -0.10556500

Name: 2-1 endo

Absolute *E* (Hartree): −490.029036

Absolute *G* (Hartree): −490.081311

Coordinates:

X Y Z

C -0.05536300 -1.01109900 1.36820900

C 0.21974300 -1.43920900 -0.06670700

C -0.38140700 0.73516500 -0.11004300

C -0.41529600 0.27780100 1.34445900

C 0.81324200 -0.11934400 -0.63508100

C -1.17546300 -1.49019100 -0.77832500

H -1.08881000 -1.91220300 -1.78240300

H -1.89411500 -2.09943000 -0.22403300

C -1.57646600 0.02588400 -0.82924200

H 0.80626500 -2.34888000 -0.19307300

H -0.34551500 1.81037900 -0.27259300

H -0.79157800 0.87180300 2.16600500

H -0.07194100 -1.67788300 2.22184800

H 0.81470400 -0.13169300 -1.74019800

H -1.61333300 0.34514500 -1.88045400

B 2.27445200 0.33222100 -0.21327200

C 2.66880900 1.85227200 -0.32123600

H 2.51231000 2.28588900 0.67977700

H 3.72664800 2.01393000 -0.55177100

H 2.05737600 2.44355000 -1.00909600

C 3.36268800 -0.72182300 0.21117000

H 4.21672000 -0.28844100 0.73993600

H 2.97374000 -1.56350900 0.79064800

H 3.76638900 -1.15547000 -0.71857900

C -2.95126800 0.32274800 -0.28478100

O -3.21675300 1.18340900 0.51923600

H -3.76240600 -0.31478700 -0.70407000

Name: 2-1 exo

Absolute *E* (Hartree): −490.032423

Absolute *G* (Hartree): −490.079196

Coordinates:

X Y Z

C 2.43014400 -1.05954400 0.09492300

C 1.11733500 -0.91450200 0.84359100

C 0.92226500 0.21427200 -1.08732900

C 2.32065500 -0.36321400 -1.04398400

C 0.08702200 -0.84925500 -0.32229600

C 1.10926300 0.57334500 1.34350500

H 0.30485000 0.74042300 2.06211700

H 2.04792100 0.86890500 1.81393600

C 0.87518000 1.37161200 0.00758200

H 1.61693900 2.15392400 -0.15460200

H 0.92951700 -1.63514600 1.63878300

H 0.56151900 0.53646500 -2.06409700

H 3.09759600 -0.16248000 -1.77153500

H 3.32026200 -1.54112300 0.48226700

H 0.12177200 -1.79240300 -0.88107400

B -1.47846800 -0.48831900 -0.06629000

C -2.12989700 -0.93335700 1.33391800

H -2.20591700 -2.02853200 1.35632700

H -1.55992400 -0.64482100 2.22471300

H -3.14841400 -0.54877200 1.45734500

C -2.41233400 -0.72901900 -1.35448000

H -2.57715800 -1.80600400 -1.48727300

H -3.40148000 -0.27074500 -1.24511700

H -1.97998000 -0.36054600 -2.29328800

O -1.51409500 1.28224700 0.02723600

C -0.48770600 1.95997700 0.01375300

H -0.61397100 3.05258400 -0.00002900

Name: 2-2 endo

Absolute *E* (Hartree): −529.328003

Absolute *G* (Hartree): −529.384890

Coordinates:

X Y Z

C 0.35111200 -0.98052300 1.48892400

C 0.54200200 -1.42971500 0.04777500

C -0.02265500 0.75269100 0.00833100

C 0.01457700 0.31602400 1.46682400

C 1.12541100 -0.12790600 -0.57005000

C -0.89240400 -1.45977000 -0.58457700

H -0.86407400 -1.89451400 -1.59105500

H -1.58447800 -2.05090400 0.02028400

C -1.27230200 0.05922300 -0.63573600

H 1.10577300 -2.35174400 -0.10035900

H 0.02083500 1.82525100 -0.17291700

H -0.31932800 0.92349400 2.29748200

H 0.35503600 -1.63919900 2.35045300

H 1.04684600 -0.15812000 -1.67841800

H -1.31011800 0.36007800 -1.70351500

B 2.61857400 0.29286500 -0.28507000

C 3.05019400 1.79932300 -0.50411600

H 3.03794000 2.30612200 0.47179400

H 4.07551900 1.90014700 -0.88484100

H 2.37421200 2.36797200 -1.15435300

C 3.73047900 -0.79093800 -0.00347700

H 4.66441600 -0.38112400 0.39449300

H 3.40345900 -1.61637500 0.63477600

H 3.98681000 -1.24807100 -0.97996900

C -2.60695500 0.46014900 -0.05375000

O -2.73829400 1.44841500 0.66803200

C -3.82009900 -0.27445200 -0.58902900

H -3.69840200 -1.35850600 -0.57078100

H -4.71017500 0.01376800 -0.02899800

H -3.96694500 0.00544600 -1.64970500

Name: 2-2 exo

Absolute *E* (Hartree): −529.342531

Absolute *G* (Hartree): −529.392916

Coordinates:

X Y Z

C 2.77890600 -0.56429000 0.09283300

C 1.48189000 -0.82201700 0.84012500

C 0.95885000 0.20828800 -1.08432100

C 2.46690200 0.07242600 -1.04309400

C 0.47907200 -1.06094900 -0.32692600

C 1.02516200 0.59081500 1.34595100

H 0.20057400 0.50194800 2.05553100

H 1.82851900 1.14848000 1.82967100

C 0.57195500 1.29075900 0.01328800

H 1.06290000 2.25150900 -0.14430800

H 1.51799400 -1.57152200 1.62989400

H 0.51886500 0.41185000 -2.06110700

H 3.14934000 0.49763400 -1.76910800

H 3.77219800 -0.76048600 0.47937000

H 0.78997700 -1.94914100 -0.88984900

B -1.12461500 -1.16309900 -0.06828400

C -1.61237300 -1.80779400 1.32418200

H -1.37374800 -2.87944400 1.32513500

H -1.14385000 -1.38572300 2.22125000

H -2.69778400 -1.73105900 1.45567500

C -1.94476600 -1.67096300 -1.36021400

H -1.77407000 -2.74550000 -1.50418600

H -3.02649100 -1.53627000 -1.24699200

H -1.64886700 -1.17886900 -2.29566500

O -1.66869500 0.48708600 0.03691700

C -0.91318300 1.46666100 0.02121100

C -1.50845800 2.84149000 0.00538800

H -1.19684400 3.35849600 -0.90858000

H -2.59520300 2.79647500 0.05444600

H -1.11461400 3.42386900 0.84436300

Name: 2-3 endo

Absolute *E* (Hartree): −565.304275

Absolute *G* (Hartree): −565.358668

Coordinates:

X Y Z

C 0.33070600 -0.95306200 1.44973900

C 0.51787000 -1.42925400 0.01633800

C -0.03247100 0.75721500 -0.06081500

C 0.00023300 0.34331000 1.40659500

C 1.11080600 -0.14176800 -0.62261700

C -0.91488500 -1.46462900 -0.61507200

H -0.89862400 -1.91737400 -1.60895900

H -1.62131900 -2.02990700 -0.00648900

C -1.27770200 0.05891100 -0.70185500

H 1.07588400 -2.35624300 -0.11322400

H 0.01984100 1.82599600 -0.25805500

H -0.31730100 0.97074700 2.22800700

H 0.34430700 -1.59406700 2.32301900

H 1.05192500 -0.18559800 -1.72535900

H -1.34976400 0.34717300 -1.75670500

B 2.60241800 0.28641300 -0.29518600

C 3.02902900 1.78970800 -0.48553400

H 2.95609200 2.26313600 0.50697900

H 4.07270000 1.91017300 -0.79346400

H 2.38885200 2.37476000 -1.15215400

C 3.68457900 -0.77924700 0.11561000

H 4.58133000 -0.34853300 0.57060000

H 3.30640700 -1.57834000 0.75911500

H 4.01743200 -1.27169500 -0.81283400

C -2.60058900 0.45272400 -0.09030600

O -2.78784400 1.33967400 0.70604600

O -3.62322300 -0.29888400 -0.57984000

H -4.43676900 0.02794300 -0.16668700

Name: 2-3 exo

Absolute *E* (Hartree): −565.307491

Absolute *G* (Hartree): −565.357125

Coordinates:

X Y Z

C 2.71563900 -0.75776600 0.08091900

C 1.40715200 -0.91597900 0.83742900

C 0.95375900 0.16228800 -1.08053200

C 2.44750900 -0.09527700 -1.05086200

C 0.38541400 -1.07492300 -0.32855200

C 1.06620200 0.52547900 1.34907300

H 0.23765200 0.50442800 2.05933800

H 1.91258700 1.01248400 1.83487500

C 0.67468200 1.26925200 0.02239500

H 1.23252800 2.18984900 -0.13799100

H 1.39085100 -1.67025700 1.62322800

H 0.52437300 0.40514000 -2.05273700

H 3.15550800 0.27879500 -1.78021200

H 3.69274000 -1.03215600 0.46078500

H 0.63806600 -1.97638800 -0.90091600

B -1.21488200 -1.11513600 -0.07461200

C -1.75251300 -1.69545600 1.31580200

H -1.60261400 -2.78372300 1.32548200

H -1.24624400 -1.30659100 2.20625600

H -2.82751900 -1.53040800 1.44718800

C -2.07954200 -1.48758900 -1.37163200

H -2.01409600 -2.56918400 -1.54991100

H -3.14284700 -1.25590200 -1.24613900

H -1.73948700 -0.99872500 -2.29264100

C -0.78547800 1.56332800 0.03294300

O -1.65912700 0.69971600 0.06610300

O -1.12130400 2.85458800 -0.00016800

H -2.09058600 2.91373600 0.00706000

Name: 2-4 endo

Absolute *E* (Hartree): −564.761593

Absolute *G* (Hartree): −564.815867

Coordinates:

X Y Z

C 0.23716900 -1.13777500 1.28120100

C 0.46379200 -1.44110500 -0.19404300

C -0.08311800 0.74227600 -0.02367900

C -0.08903800 0.15817800 1.38185400

C 1.08224300 -0.09198000 -0.65710400

C -0.95114600 -1.37534100 -0.86806000

H -0.88436300 -1.69184400 -1.91544700

H -1.69460000 -2.00418300 -0.37726300

C -1.33619800 0.12980600 -0.74174400

H 1.02940700 -2.35108400 -0.41170300

H -0.03108600 1.82655100 -0.08747500

H -0.48135600 0.67276500 2.24874500

H 0.17511300 -1.88567200 2.06430100

H 1.03962600 0.00842000 -1.75689000

H -1.40359600 0.57884400 -1.74076500

B 2.54376700 0.29909100 -0.22072600

C 3.02512000 1.79587500 -0.39500300

H 2.89997600 2.28123100 0.58582700

H 4.08958300 1.88824300 -0.64216900

H 2.43568400 2.38322400 -1.10514400

C 3.59041800 -0.76482700 0.30046400

H 4.41493100 -0.33260900 0.87880100

H 3.14041500 -1.58236500 0.86920100

H 4.04784100 -1.22245500 -0.59246900

C -2.70528700 0.44792000 -0.04565700

O -2.76525000 1.56946300 0.51812900

O -3.59726800 -0.42479800 -0.16104100

Name: 2-4 exo

Absolute *E* (Hartree): −564.808985

Absolute *G* (Hartree): −564.856891

Coordinates:

X Y Z

C 2.65934700 -0.88252100 0.09346400

C 1.33626500 -0.94872300 0.84023100

C 0.96646200 0.15308000 -1.07945100

C 2.44034100 -0.20248200 -1.04136400

C 0.30759300 -1.03833000 -0.32951700

C 1.08275700 0.51695900 1.33496900

H 0.25609400 0.54798300 2.04752600

H 1.96235000 0.95027800 1.81954500

C 0.71702200 1.26331200 0.01033800

H 1.31485000 2.16084600 -0.14842600

H 1.26363600 -1.70203700 1.62675800

H 0.55901800 0.41278700 -2.05807300

H 3.18691400 0.13275200 -1.75473200

H 3.62033900 -1.20204500 0.48576000

H 0.47942300 -1.96997900 -0.88564000

B -1.30297500 -0.83531900 -0.06087800

C -1.84185100 -1.54465200 1.31012900

H -1.69439400 -2.63442700 1.27846400

H -1.34353800 -1.18317000 2.22051300

H -2.91811900 -1.37175600 1.44834500

C -2.15557700 -1.34647900 -1.36234300

H -2.08782800 -2.43753000 -1.48597100

H -3.22071100 -1.09627200 -1.26457400

H -1.80768300 -0.89509000 -2.30320500

C -0.75650100 1.67719100 0.01788800

O -1.62707800 0.70736800 0.05567500

O -1.07175100 2.86514700 0.00832900

Name: 2-5 endo

Absolute *E* (Hartree): −545.415524

Absolute *G* (Hartree): −545.471048

Coordinates:

X Y Z

C 0.32841800 -0.96525900 1.44138100

C 0.52302200 -1.43732100 0.00738700

C -0.03171700 0.74753400 -0.06671000

C -0.00528500 0.33052800 1.39877500

C 1.11495200 -0.14780200 -0.62638400

C -0.90962100 -1.46951500 -0.62916600

H -0.88261500 -1.92493900 -1.62331000

H -1.60351500 -2.04583500 -0.01340400

C -1.28021000 0.05204100 -0.70819700

H 1.08091700 -2.36448700 -0.12271300

H 0.01581000 1.81640300 -0.25987800

H -0.33114400 0.95611200 2.21825600

H 0.33763100 -1.60935900 2.31251500

H 1.06090200 -0.18790100 -1.72945700

H -1.33894700 0.35204900 -1.76138900

B 2.60355200 0.28196900 -0.28820800

C 3.03021700 1.78469300 -0.48279300

H 2.94423100 2.26354200 0.50607700

H 4.07718600 1.90575600 -0.77923600

H 2.39598800 2.36454000 -1.15950000

C 3.68472500 -0.77926800 0.13786900

H 4.57083100 -0.34430800 0.60956000

H 3.30136600 -1.58340100 0.77184500

H 4.03718000 -1.26467700 -0.78711700

C -2.60657800 0.48470500 -0.08904800

O -2.72240600 1.48873700 0.59388400

N -3.68598100 -0.29602400 -0.40323700

H -4.58867300 -0.03441800 -0.03846200

H -3.60132500 -1.14867400 -0.92984300

Name: 2-5 exo

Absolute *E* (Hartree): −545.426312

Absolute *G* (Hartree): −545.475735

Coordinates:

X Y Z

C 2.73141100 -0.72313300 0.08363900

C 1.42187700 -0.89936000 0.83419200

C 0.96320600 0.17995000 -1.08105100

C 2.45994700 -0.05844600 -1.04631900

C 0.40193700 -1.06264100 -0.33333900

C 1.05985100 0.53523000 1.34977600

H 0.23099700 0.49850500 2.05890000

H 1.89939800 1.03388900 1.83686600

C 0.65158000 1.27068000 0.02565200

H 1.20201100 2.20029500 -0.13068700

H 1.41048100 -1.65714000 1.61666700

H 0.53378200 0.42040700 -2.05424100

H 3.16750900 0.32242000 -1.77308700

H 3.71059300 -0.99016100 0.46409000

H 0.65471600 -1.96424200 -0.90381000

B -1.20756300 -1.06737400 -0.07061300

C -1.71810500 -1.71633000 1.31206000

H -1.53030000 -2.79810100 1.30068600

H -1.22826600 -1.32581900 2.21240400

H -2.79844800 -1.58962300 1.44942900

C -2.05002900 -1.52148500 -1.36922200

H -1.93694900 -2.60137000 -1.53010700

H -3.12344200 -1.33090500 -1.25331100

H -1.72990900 -1.03071900 -2.29775000

C -0.82487500 1.52984600 0.03132600

O -1.65541900 0.59958900 0.06080000

N -1.26747900 2.79934400 0.00271000

H -2.26129700 2.97562900 0.01333900

H -0.62816700 3.57581800 -0.00197800

# **Cartesian Coordinates, Imaginary Frequencies, Absolute Internal Energies (*E*), and Absolute Gibbs Free Energies (*G*) of TS Structures at 298.15 K**

Name: 1-1 endo

Imaginary Frequency: 413.99 cm^−1^

Absolute *E* (Hartree): −451.872925

Absolute *G* (Hartree): −451.923211

Coordinates:

X Y Z

C -1.18526800 -1.30349900 -0.88764300

C -1.62011100 -0.92060200 0.38266300

C -0.60216700 0.88772900 -0.67995300

C -0.54588900 -0.21251900 -1.51363800

C -1.66356400 0.60445200 0.38072800

C 0.11650900 -1.02485500 1.55584100

C 0.86761800 0.10495000 1.20885700

H -2.32899200 -1.49423700 0.96650100

H -0.25146800 1.88324800 -0.91937000

H -0.01995800 -0.26044800 -2.45800000

H -1.23060900 -2.30902400 -1.28565100

H -0.43508700 -1.03109000 2.49212500

B 2.01525600 0.16951900 0.18499900

H 0.66081700 1.00494000 1.78746300

C 2.50837000 -1.12266500 -0.58755300

H 1.75387900 -1.90516100 -0.71081000

H 2.93911000 -0.90186000 -1.56999400

H 3.32094200 -1.57263200 0.00320400

C 2.82081700 1.51969400 -0.01709900

H 3.86476300 1.37844000 0.29586500

H 2.87730800 1.80131700 -1.07627200

H 2.42564200 2.37437800 0.54093500

O -2.97870600 0.99774100 -0.05816800

H -2.98322500 1.95407600 -0.16838700

H -1.46635000 1.05846800 1.35063400

H 0.44871300 -2.00604600 1.23889200

Name: 1-1 exo

Imaginary Frequency: 415.93 cm^−1^

Absolute *E* (Hartree): −451.876231

Absolute *G* (Hartree): −451.924691

Coordinates:

X Y Z

C 2.51380300 0.60370300 0.46311800

C 1.93895000 -0.67447700 0.43216500

C 0.80791900 0.72442200 -1.04803400

C 1.79650400 1.45299800 -0.39963800

C 1.16823700 -0.72361600 -0.88367500

H 1.93573500 -0.92048000 -1.65450200

C 0.20706700 -0.45804600 1.64772800

H 0.78018700 -0.19681200 2.52998700

C -0.57363300 0.50361500 1.00450200

H -0.33258100 1.53797000 1.24461100

H 2.41053900 -1.55404000 0.85701900

H 0.15229800 1.08658500 -1.82744100

H 1.94075200 2.52219900 -0.48393300

H 3.30396400 0.91895700 1.13225800

O 0.10452400 -1.63544700 -1.04569500

H 0.46769000 -2.50221600 -1.25061500

B -1.90900800 0.28051300 0.25014100

C -2.65965100 -1.10864100 0.19917700

H -2.08725200 -1.95971700 0.57155900

H -3.57565100 -1.03228900 0.80352200

H -2.99654100 -1.34535200 -0.81690100

C -2.63908000 1.53256600 -0.39472800

H -2.83849600 1.35858300 -1.46068600

H -3.62909900 1.67609300 0.05881600

H -2.09223800 2.47634600 -0.30526000

H -0.09116100 -1.49995300 1.59273500

Name: 1-2 endo

Imaginary Frequency: 423.11 cm^−1^

Absolute *E* (Hartree): −431.985822

Absolute *G* (Hartree): −432.036330

Coordinates:

X Y Z

C -1.14442800 -1.31232900 -0.89724100

C -1.59294300 -0.94081300 0.37688800

C -0.61877000 0.88820400 -0.67110500

C -0.52598500 -0.20923800 -1.50957500

C -1.68239800 0.58638300 0.37585700

C 0.12470100 -1.02872900 1.53784700

C 0.87360100 0.10848900 1.19962300

H -2.28999600 -1.54434100 0.94801600

H -0.28432000 1.89026300 -0.90588200

H 0.01395500 -0.23888400 -2.44675700

H -1.16536700 -2.31768500 -1.29791900

H -0.41922600 -1.04167700 2.47893200

B 2.03261600 0.18292200 0.19289100

H 0.65584800 1.00548700 1.77844200

C 2.54245500 -1.10143500 -0.58304000

H 1.79602900 -1.89071900 -0.71193300

H 2.97343500 -0.87177000 -1.56351600

H 3.35840200 -1.54521000 0.00766900

C 2.83020300 1.54009900 0.00132000

H 3.87754900 1.40428800 0.30489900

H 2.87616000 1.83255200 -1.05566400

H 2.43138700 2.38637600 0.56954300

H -1.46567400 1.02606000 1.35062300

H 0.47462400 -2.00662000 1.22852900

N -3.00883500 1.00682800 -0.13412000

H -3.73171700 0.74818400 0.53073700

H -3.04094000 2.01750500 -0.22478400

Name: 1-2 exo

Imaginary Frequency: 474.48 cm^−1^

Absolute *E* (Hartree): −431.997689

Absolute *G* (Hartree): −432.043691

Coordinates:

X Y Z

C -2.69701900 -0.33876100 -0.02573400

C -1.59892100 -0.84967100 0.69062400

C -1.05997400 1.24901800 -0.13624600

C -2.34239300 0.90873800 -0.55858400

C -0.75638100 0.36528500 1.04335200

H -1.20090600 0.80847100 1.94847500

C -0.35375700 -1.48092200 -0.89669400

H -1.09517900 -1.85546200 -1.59570900

C 0.39234000 -0.33220400 -1.16880300

H 0.23652500 0.16305100 -2.12286100

H -1.66580800 -1.70603900 1.35334500

H -0.58622900 2.20611400 -0.29930700

H -2.93441100 1.47733000 -1.26400000

H -3.59363300 -0.89534000 -0.26295600

B 1.60108900 0.04554500 -0.17516500

N 0.69584200 0.13982200 1.29251800

H 1.07335400 0.90631500 1.84638200

H 0.82800300 -0.70459700 1.84681000

C 2.70310300 -1.12407200 0.07307900

H 3.40181100 -0.87676900 0.88538700

H 2.27442300 -2.10926300 0.30209800

H 3.31685700 -1.26033000 -0.82453900

C 2.29488300 1.49300300 -0.40006300

H 3.00958800 1.74789800 0.39578400

H 2.87890300 1.47426400 -1.32834200

H 1.60098000 2.33653500 -0.49515500

H 0.08809000 -2.26114800 -0.28279700

Name: 1-3 endo

Imaginary Frequency: 292.98 cm^−1^

Absolute *E* (Hartree): −564.732450

Absolute *G* (Hartree): −564.788862

Coordinates:

X Y Z

C -0.37645000 -1.59178000 -0.53580800

C -0.77328100 -1.02387500 0.71458500

C -0.51965900 0.64384000 -0.84898500

C -0.12974700 -0.56320400 -1.43819600

C -1.24532800 0.36419800 0.38162600

C 0.83501300 -0.66954200 1.63760300

C 1.57724900 0.42412300 1.09033400

H -1.33169200 -1.63041700 1.42070000

H -0.44667800 1.62318800 -1.30234900

H 0.30857100 -0.67078500 -2.42210300

H -0.19268500 -2.64437600 -0.71252800

H 0.37319900 -0.50172300 2.61090800

B 2.71467700 0.35036800 0.11282000

H 1.25782100 1.40960000 1.43311000

C 3.28164900 -1.03161500 -0.46346000

H 2.63529000 -1.89725500 -0.28607000

H 3.47229400 -0.97621600 -1.54405000

H 4.25499300 -1.25859000 -0.00159700

C 3.48130400 1.67696600 -0.35479200

H 4.55014100 1.63055300 -0.09690600

H 3.44965000 1.79234700 -1.44794000

H 3.07572600 2.59512800 0.08507200

H -1.18203400 1.11656100 1.16037800

C -2.93898200 0.42303400 -0.02674800

O -3.42805100 -0.65025200 -0.36032300

O -3.35427700 1.57464800 0.11055700

H 1.31003300 -1.64790100 1.60045700

Name: 1-3 exo

Imaginary Frequency: 457.94 cm^−1^

Absolute *E* (Hartree): −564.749499

Absolute *G* (Hartree): −564.799401

Coordinates:

X Y Z

C -2.81858600 -0.65299300 0.29770300

C -2.01689500 0.42860600 0.67880000

C -1.02639200 -0.63203900 -1.11756800

C -2.21080300 -1.30069200 -0.78971000

C -1.11622500 0.74080300 -0.50075800

H -1.73364400 1.35586000 -1.17706400

C -0.34822500 -0.73572000 1.64229600

C 0.34915900 -1.21643600 0.53258100

H 0.22846800 -2.27760100 0.31636800

H -2.28835400 1.18232800 1.40740500

H -0.43666600 -0.84035200 -1.99798200

C 0.12236700 1.61776100 -0.23300300

B 1.67528400 -0.44066400 -0.01657300

H -2.53048300 -2.24503800 -1.21564400

H -3.66976200 -1.02556500 0.85620900

O -0.06531000 2.82836600 -0.13071700

O 1.30192000 1.08095200 -0.14640500

H -0.97297600 -1.38012100 2.25372900

C 2.86843000 -0.48267500 1.10721700

H 3.21402800 -1.51137400 1.28539500

H 3.74103100 0.10261000 0.78356500

H 2.54259600 -0.07943700 2.07461400

C 2.25726200 -0.96607600 -1.45319500

H 3.20086000 -0.45984800 -1.69901300

H 2.47786300 -2.04334000 -1.41543300

H 1.58906000 -0.80803300 -2.30968900

H 0.00812400 0.15544200 2.15120700

Name: 2-1 endo

Imaginary Frequency: 451.97 cm^−1^

Absolute *E* (Hartree): −489.984013

Absolute *G* (Hartree): −490.036834

Coordinates:

X Y Z

C -0.92537600 -0.76933300 0.81019800

C -0.33271300 0.31917800 -1.34311800

C 0.30031800 1.67626700 0.41383800

C -0.56732200 0.33416300 1.60805700

H -1.37006000 0.92231500 2.04003600

H 0.27830800 0.22101900 2.27751800

C 0.90684100 0.68570100 -0.57490800

H 1.49571700 1.32824200 -1.27035500

C -1.24160400 1.37163700 -1.24964000

C -0.83268200 2.23226100 -0.22902400

H -0.39288500 -0.49021500 -2.05766200

H 0.92038700 2.26316600 1.08235400

B 1.99398700 -0.39427000 -0.16931900

C 2.90113800 -0.18731100 1.09972700

H 3.93816400 -0.49078400 0.91939100

H 2.89762700 0.81808600 1.52825100

H 2.53683500 -0.87051900 1.88215000

C 2.27738900 -1.61732600 -1.11659000

H 2.72742400 -2.46818600 -0.59611200

H 1.42233300 -1.97186600 -1.69676100

H 3.03183600 -1.28483900 -1.84767700

H -1.38795400 3.09094000 0.12724200

H -2.16552000 1.44995500 -1.80758200

C -2.30352200 -1.04559500 0.44153800

O -2.71991100 -2.09392400 -0.02258100

H -0.23343900 -1.59256900 0.66167100

H -3.00423400 -0.19987000 0.62432000

Name: 2-1 exo

Imaginary Frequency: 250.74 cm^−1^

Absolute *E* (Hartree): −490.005559

Absolute *G* (Hartree): −490.054058

Coordinates:

X Y Z

C 0.84531000 1.84271900 -0.43488400

C 0.84687100 -1.16946800 -1.05491300

C 1.17563700 -0.60240000 1.16179800

C 1.04217900 1.53195900 0.90796600

H 1.65691200 2.26114700 -1.01900800

H 1.96533900 1.84070400 1.38198900

H 0.18341600 1.48043800 1.56135200

C 0.08872000 -1.01038400 0.20220500

H -0.24307700 -2.01533000 0.51576000

C 2.20223900 -1.04135100 -0.83548400

C 2.41348200 -0.75961900 0.53533800

H 0.38948500 -1.40705800 -2.00693000

H 1.04101300 -0.59866400 2.23618400

C -0.38109400 1.69538500 -1.09444400

B -1.45392400 -0.31394700 0.15131600

C -2.04763200 0.00732300 1.62199200

H -3.02992300 0.48629500 1.53789700

H -2.20340400 -0.92571600 2.17823100

H -1.44178800 0.65491600 2.26756100

C -2.44972000 -1.24716300 -0.72099500

H -3.42383200 -0.76213800 -0.84910800

H -2.07501800 -1.47202500 -1.72751800

H -2.63579800 -2.20839200 -0.22519600

H 3.37869600 -0.61598900 1.00546500

H 2.97854800 -1.13113000 -1.58423200

O -1.39709600 1.03403500 -0.71472800

H -0.51012700 2.22839400 -2.04267100

Name: 2-2 endo

Imaginary Frequency: 474.59 cm^−1^

Absolute *E* (Hartree): −529.288597

Absolute *G* (Hartree): −529.345082

Coordinates:

X Y Z

C 1.54933200 0.52422000 0.88312400

C -0.29335800 0.58095100 -0.64452100

C -0.61317300 -1.19500600 0.77646200

C 1.13284500 -0.63480200 1.57199600

H 1.71772000 -1.54101100 1.46703800

H 0.74515800 -0.50152000 2.57698000

C -1.13651600 0.20542000 0.54539900

C 0.00331500 -0.60029200 -1.34086100

C -0.22498200 -1.68547800 -0.49947900

H -0.23116400 1.57814700 -1.05961300

H -1.04405500 -1.84907000 1.52759600

H -0.00390700 -2.72011800 -0.72929200

H 0.45194400 -0.64472600 -2.32471700

C 2.67954300 0.59525200 -0.05168800

O 3.10668000 1.67586100 -0.44172100

H 1.25054000 1.49792400 1.25398500

H -1.00780200 0.88249000 1.39236300

B -2.65999600 0.35747000 0.04163100

C -3.53959300 -0.86177200 -0.40609700

H -4.41084000 -0.57905700 -1.00415300

H -2.97941900 -1.64140200 -0.92821900

H -3.92954200 -1.33677200 0.50813300

C -3.28244000 1.80327300 0.05583000

H -3.59347600 2.07956400 -0.96079200

H -4.21050600 1.80804800 0.64328700

H -2.63388100 2.59528900 0.43919800

C 3.34884900 -0.69535500 -0.50147100

H 3.97467000 -1.08478700 0.30856700

H 2.62356300 -1.47212500 -0.75632100

H 3.98460600 -0.48358700 -1.36059800

Name: 2-2 exo

Imaginary Frequency: 284.04 cm^−1^

Absolute *E* (Hartree): −529.310748

Absolute *G* (Hartree): −529.363021

Coordinates:

X Y Z

C 0.31542600 1.67079100 0.71979300

C 1.05242600 -0.23515600 -1.49154700

C 1.68340900 -0.87554700 0.63551300

C 0.88850200 0.77652400 1.62924700

H 0.84053200 2.58330600 0.46565700

H 1.75620300 1.10705500 2.18684600

H 0.23352800 0.10657400 2.16925800

C 0.58516500 -1.08066500 -0.37612400

H 0.65966200 -2.13033200 -0.71006000

C 2.33354700 0.22856500 -1.26126100

C 2.75306300 -0.22207200 0.00832900

H 0.48144600 -0.06273700 -2.39515600

H 1.80476300 -1.51537200 1.50111700

C -0.95946500 1.48766800 0.15686900

B -1.05846900 -1.11116500 0.01219800

C -1.35374600 -1.92635600 1.37959700

H -2.42238600 -1.88874500 1.62100800

H -1.09972000 -2.98661000 1.25461100

H -0.82097100 -1.58336000 2.27513600

C -1.91065200 -1.68641900 -1.24151300

H -2.98613800 -1.62334300 -1.04098300

H -1.73309200 -1.14595100 -2.18039800

H -1.68453600 -2.74327200 -1.43274700

H 3.71525600 -0.01617600 0.46146300

H 2.91577700 0.84592900 -1.93307800

O -1.60239000 0.38273900 0.15713500

C -1.69465800 2.65309900 -0.45214100

H -2.01338400 2.40410100 -1.46737200

H -2.60147900 2.83898000 0.13129800

H -1.09210100 3.56127500 -0.46524700

Name: 2-3 endo

Imaginary Frequency: 460.87 cm^−1^

Absolute *E* (Hartree): −565.258899

Absolute *G* (Hartree): −565.314478

Coordinates:

X Y Z

C 1.57787500 0.51889000 0.90797800

C -0.29353900 0.61627000 -0.64251800

C -0.58412900 -1.18294800 0.75772700

C 1.13537600 -0.63928000 1.57866500

H 1.72192200 -1.54192400 1.46437300

H 0.73784700 -0.50544700 2.57969200

C -1.12550800 0.21435900 0.54509200

C 0.03482400 -0.54798300 -1.34836300

C -0.17574300 -1.64714900 -0.52091200

H -0.24239900 1.62097500 -1.04090400

H -1.01508700 -1.85600900 1.49197900

H 0.08028600 -2.67096700 -0.76017300

H 0.49813400 -0.57410000 -2.32544400

C 2.67585800 0.53484900 -0.04590100

O 3.21074800 1.52853300 -0.50111200

H 1.32390200 1.50229600 1.27922900

O 3.09468600 -0.71827200 -0.41782800

H 3.81670400 -0.57827200 -1.04668100

B -2.65138200 0.35104200 0.04511100

C -3.50730600 -0.87335500 -0.43303100

H -3.89877700 -1.37007700 0.46894400

H -4.37737600 -0.59306300 -1.03398300

H -2.93107900 -1.63514100 -0.96383200

C -3.30134500 1.78415200 0.09301700

H -3.62693100 2.07595000 -0.91455700

H -4.22397300 1.75806600 0.68857300

H -2.66535500 2.58041300 0.48863000

H -1.00255000 0.88317600 1.39958700

Name: 2-3 exo

Imaginary Frequency: 309.87 cm^−1^

Absolute *E* (Hartree): −565.265164

Absolute *G* (Hartree): −565.315852

Coordinates:

X Y Z

C 0.52300900 1.62986900 0.61410300

C 1.01343500 -0.34421200 -1.45447700

C 1.51805000 -1.00752400 0.69792600

C 0.97633700 0.70155200 1.56676700

H 1.17080700 2.43265900 0.28180000

H 1.87174800 0.96167900 2.11829600

H 0.22615100 0.20142700 2.16687100

C 0.42090700 -1.11229900 -0.34032600

H 0.41488000 -2.16938400 -0.66325200

C 2.35786100 -0.09502500 -1.22904300

C 2.69260800 -0.57367800 0.04818400

H 0.48045300 -0.09405400 -2.36353000

H 1.54493700 -1.65599900 1.56614500

C -0.79283000 1.58717900 0.10493500

B -1.21101200 -0.96749600 0.00497600

C -1.62601300 -1.70459100 1.38164600

H -2.68501000 -1.53319700 1.60555900

H -1.49882500 -2.79044400 1.28627300

H -1.06489600 -1.40275000 2.27504600

C -2.10722400 -1.43198100 -1.25824800

H -3.16996300 -1.24074500 -1.07366500

H -1.85344500 -0.91854300 -2.19434800

H -2.00556100 -2.50883600 -1.44498100

H 3.67358400 -0.51351600 0.50427100

H 3.03274600 0.40420800 -1.91233500

O -1.57942400 0.60689500 0.15319300

O -1.32166500 2.68474900 -0.48016200

H -0.73067500 3.43607900 -0.35008900

Name: 2-4 endo

Imaginary Frequency: 523.26 cm^−1^

Absolute *E* (Hartree): −564.709740

Absolute *G* (Hartree): −564.764892

Coordinates:

X Y Z

C 1.56636100 0.79294200 -0.59953600

C -0.02134400 -0.70059000 -0.55772600

C -0.76326500 0.80712300 1.00876200

C 1.11296800 1.69435600 0.37161800

H 1.63449400 1.70009700 1.32161300

H 0.64769400 2.62949300 0.07154600

C -1.07861400 0.36526800 -0.41744100

C 0.20014700 -1.24100900 0.72381100

C -0.26648600 -0.33846700 1.67365300

H 0.15634300 -1.24548000 -1.47713700

H -1.30224900 1.60578600 1.50857600

H -0.06953600 -0.39974100 2.73676600

H 0.81012400 -2.10754000 0.93546100

C 2.80237700 -0.09505300 -0.36288500

O 3.33602200 -0.54398600 -1.40812600

H 1.36805200 1.01279500 -1.64548200

O 3.13200400 -0.27577400 0.83532700

B -2.60350600 -0.08095000 -0.35337000

C -3.70099000 1.04055100 -0.59169300

H -4.12601400 0.89189000 -1.59673300

H -4.54678000 0.95309000 0.10080500

H -3.31937600 2.06567100 -0.55534500

C -3.08564100 -1.56891900 -0.15731000

H -4.10256600 -1.65707800 0.24192900

H -3.09601000 -2.04292500 -1.15163200

H -2.39772700 -2.16147900 0.44940900

H -0.97619300 1.18102800 -1.13804600

Name: 2-4 exo

Imaginary Frequency: 481.93 cm^−1^

Absolute *E* (Hartree): −564.757278

Absolute *G* (Hartree): −564.806812

Coordinates:

X Y Z

C 0.54245000 1.53113600 0.41962100

C 1.02739500 0.00283100 -1.29798300

C 1.49672600 -1.07054900 0.66916800

C 0.95543200 0.75436600 1.50690600

H 1.16287400 2.34393000 0.06499100

H 1.87108400 1.02157300 2.02273800

H 0.19512000 0.30979600 2.13747000

C 0.40750300 -1.01340200 -0.38292100

H 0.49170300 -1.98213700 -0.91462600

C 2.41629200 0.00367000 -1.11711900

C 2.71419700 -0.68958300 0.05952200

H 0.52347700 0.41042700 -2.16630000

H 1.44903600 -1.75962200 1.50641200

C -0.90065700 1.65856300 0.07820300

B -1.21252300 -0.89754500 -0.03352800

C -1.62718600 -1.76513500 1.28972500

H -2.70488300 -1.67991600 1.48441900

H -1.41223100 -2.83524400 1.15251000

H -1.11593000 -1.45480700 2.21182100

C -2.07955000 -1.38341400 -1.33809600

H -3.15344800 -1.22661400 -1.17225100

H -1.81823500 -0.83039700 -2.25185200

H -1.93534100 -2.45254600 -1.55536000

H 3.69633700 -0.76326700 0.51477400

H 3.13010900 0.55201800 -1.72258800

O -1.64219400 0.58313300 0.19956700

O -1.36188900 2.74982000 -0.25790100

Name: 2-5 endo

Imaginary Frequency: 497.59 cm^−1^

Absolute *E* (Hartree): −545.366479

Absolute *G* (Hartree): −545.422960

Coordinates:

X Y Z

C 1.55152500 0.51626700 0.86447600

C -0.25721700 0.56920800 -0.61102700

C -0.63436100 -1.20663900 0.79496400

C 1.14655700 -0.60242400 1.61406000

H 1.72408200 -1.51808300 1.56400600

H 0.73377900 -0.42381200 2.60090300

C -1.13317500 0.19908700 0.56076100

C 0.02861600 -0.61710100 -1.30753100

C -0.22090800 -1.70015000 -0.46839800

H -0.19983900 1.56198500 -1.03774100

H -1.06263900 -1.85306800 1.55362400

H -0.01113300 -2.73830600 -0.69509800

H 0.48937000 -0.66654500 -2.28526600

C 2.69011700 0.54733500 -0.07851900

O 3.17964300 1.60243100 -0.46643100

H 1.29674800 1.50883300 1.21231700

N 3.21421300 -0.66488800 -0.47944300

H 2.67804400 -1.51239000 -0.38705200

H 3.87847800 -0.62405600 -1.23746900

H -1.01253500 0.86919800 1.41452500

B -2.64129500 0.37482400 0.02374100

C -3.24970100 1.82679100 0.05367500

H -3.59048200 2.11374600 -0.94971500

H -4.15892400 1.83256300 0.67091100

H -2.58554800 2.61067400 0.42709500

C -3.52566200 -0.82608500 -0.46427600

H -4.37821400 -0.52291200 -1.07896100

H -2.96253500 -1.60451700 -0.98485300

H -3.94355400 -1.31039000 0.43251000

Name: 2-5 exo

Imaginary Frequency: 337.00 cm^−1^

Absolute *E* (Hartree): −545.386587

Absolute *G* (Hartree): −545.437453

Coordinates:

X Y Z

C 0.46312200 1.60265600 0.61877900

C 1.02257000 -0.16423000 -1.43341300

C 1.58079200 -0.94637500 0.66004100

C 0.96991800 0.68520400 1.55931100

H 1.05895800 2.44814600 0.29831800

H 1.85232900 0.98969200 2.10979800

H 0.24626100 0.15628300 2.16856600

C 0.48160100 -1.05562300 -0.38045100

H 0.56847400 -2.08192200 -0.78364900

C 2.37193600 0.08930300 -1.22293300

C 2.74070200 -0.45793400 0.01230000

H 0.46815500 0.13526300 -2.31433600

H 1.64086700 -1.63927800 1.49217500

C -0.88002200 1.53590000 0.16447600

B -1.14236500 -1.03344200 -0.01371600

C -1.49464000 -1.87382500 1.32371100

H -2.56260400 -1.79768300 1.55965800

H -1.28287600 -2.94072000 1.17621200

H -0.95221400 -1.57001200 2.22808600

C -2.02394500 -1.49504700 -1.29425600

H -3.09586800 -1.38211400 -1.09424900

H -1.80958100 -0.92303800 -2.20704600

H -1.85714300 -2.55271500 -1.53567500

H 3.72692300 -0.39820700 0.45713600

H 3.02044100 0.64992400 -1.88416400

O -1.61406400 0.49275000 0.22699900

N -1.46669900 2.64109600 -0.37483500

H -1.08582300 3.55646700 -0.20320300

H -2.43726400 2.56728800 -0.63657900

# **Cartesian Coordinates, Absolute Internal Energies (*E*), and Absolute Gibbs Free Energies (*G*) of MCs at 298.15 K**

Name: 1-1

Absolute *E* (Hartree): −451.903799

Absolute *G* (Hartree): −451.968616

Coordinates:

X Y Z

C 2.64217400 -0.76718500 -0.10111600

H 3.61980700 -1.27246000 -0.20726100

O 1.58803800 -1.70995600 -0.29427600

H 1.70028000 -2.43041700 0.33439900

B -2.71981400 -0.46356200 -0.03832700

C -3.08355900 -0.70585700 1.47663000

H -3.32329000 0.18242500 2.06615400

H -3.95820000 -1.37147100 1.51793900

H -2.28328300 -1.25318900 1.98894100

C -2.08535400 -1.63602500 -0.87126500

H -0.99091200 -1.51048100 -0.81953900

H -2.29791500 -2.63505500 -0.47756500

H -2.33999000 -1.61084700 -1.93613600

C -3.48784700 1.99641900 -0.15672300

H -3.78890400 1.99159600 0.88698300

H -3.64703300 2.93081200 -0.68919100

C -2.95746300 0.91346500 -0.74321700

H -2.68597600 1.01248500 -1.79573200

C 2.65516200 -0.01381000 1.21603400

H 2.65871400 -0.49666300 2.18444400

C 2.72842100 1.30210300 0.96252100

H 2.80239600 2.09402200 1.69709900

C 2.59371300 0.34059800 -1.12563700

H 2.52870200 0.15479200 -2.18824900

C 2.68978600 1.52284300 -0.49844300

H 2.73017500 2.49661000 -0.96915200

Name: 1-2

Absolute *E* (Hartree): −432.027389

Absolute *G* (Hartree): −432.079558

Coordinates:

X Y Z

C -1.20972500 -0.19127500 -1.00449600

H -1.62141900 -0.28444200 -2.02433400

B 1.39283400 -0.44449500 0.21716100

N 0.24115400 -0.54417600 -1.08443200

H 0.31444300 -1.50528200 -1.41851600

H 0.66682400 0.03121400 -1.81099000

C 2.70731400 -1.08233600 -0.49770100

H 2.56655000 -2.13359600 -0.78884300

H 3.05030900 -0.54434400 -1.39320400

H 3.54982900 -1.07440400 0.20222700

C 0.83835700 -1.34216400 1.44349400

H 0.62313300 -2.38304800 1.16231300

H 1.60886600 -1.39230600 2.22271500

H -0.06193100 -0.94052800 1.92078500

C 2.48794400 1.95979900 0.20103100

H 2.51638500 3.00213100 0.51170700

H 3.32329300 1.63178300 -0.41338700

C 1.50841700 1.12409400 0.56844200

H 0.73297900 1.55826200 1.20565200

C -1.54157500 1.20656200 -0.54004000

H -1.07352700 2.09713800 -0.93337100

C -2.54193400 1.13208400 0.35459800

H -3.04124100 1.97113600 0.82091000

C -2.06630000 -1.06739600 -0.12221500

H -2.05553400 -2.14793000 -0.15239100

C -2.86808600 -0.27920600 0.61327100

H -3.63768100 -0.61505100 1.29563700

Name: 1-3

Absolute *E* (Hartree): −564.782706

Absolute *G* (Hartree): −564.838410

Coordinates:

X Y Z

C -1.29648600 -0.34228200 0.38626600

H -0.84017400 -0.50724300 1.37016100

C -0.36552300 -1.04093900 -0.64992000

B 1.96024500 -0.11238800 0.37249400

O -0.86637600 -1.77123900 -1.49612400

O 0.90681500 -0.81507500 -0.57437900

C 3.28606400 -1.06472400 0.22072400

H 4.15580400 -0.63726800 0.73980300

H 3.11196500 -2.06316200 0.64713100

H 3.56433000 -1.20080700 -0.83119700

C 1.51676400 -0.04184000 1.95292200

H 1.19855200 -1.01968300 2.34323700

H 2.39799700 0.24832500 2.54216100

H 0.73382000 0.68695000 2.19691000

C 2.27231300 1.36028100 -0.21303800

H 2.95967600 1.96452900 0.39285100

C 1.84763300 1.93493100 -1.34652100

H 2.15668600 2.93430300 -1.65314800

H 1.17375400 1.41490200 -2.02366000

C -2.71970900 -0.82354000 0.41206600

H -2.99318300 -1.86534600 0.50089800

C -3.55352000 0.22708700 0.28341200

H -4.63682800 0.18779100 0.27592300

C -2.76013200 1.45406100 0.13037400

H -3.17606900 2.44581500 -0.00449100

C -1.44837000 1.14513000 0.17706100

H -0.60526900 1.81435700 0.08491100

Name: 2-1

Absolute *E* (Hartree): −490.007712

Absolute *G* (Hartree): −490.060724

Coordinates:

X Y Z

C 0.05169500 -1.19403600 0.09238400

H -0.31582700 -2.23119800 0.21443700

C -0.39048000 1.89674700 -0.94996100

B -1.42307200 -0.44472500 0.18112400

C -1.97849800 -0.13888000 1.65556900

H -2.89421400 0.46154500 1.62351200

H -2.24864000 -1.08716600 2.13823700

H -1.28287900 0.36179400 2.33796100

C -2.50175700 -1.10930200 -0.80943800

H -3.42510000 -0.52283100 -0.85790100

H -2.14301600 -1.23695600 -1.83726500

H -2.77653400 -2.10723500 -0.44339800

O -1.30781100 1.13273900 -0.62101000

H -0.58672100 2.53842100 -1.82237900

C 1.08881500 1.81403800 1.00163400

H 0.28626800 1.46599600 1.63531800

H 2.03889000 2.02633900 1.47521200

C 0.88524200 2.08267400 -0.30171200

H 1.66250400 2.53783400 -0.90634700

C 1.13480200 -1.02327900 1.11819500

H 0.96380200 -1.04921700 2.18660600

C 2.34818600 -0.95631200 0.50390100

H 3.30942200 -0.86786600 0.99529100

C 2.15132400 -1.02396800 -0.93006600

H 2.94330900 -0.98155700 -1.66753900

C 0.82280600 -1.16055700 -1.18766100

H 0.37376600 -1.26893200 -2.16636100

Name: 2-2

Absolute *E* (Hartree): −529.326733

Absolute *G* (Hartree): −529.396483

Coordinates:

X Y Z

C -2.26081600 0.35577500 -0.84418100

H -2.11904300 0.90499500 -1.78149400

C 3.29804800 0.01932100 -0.36420700

B -1.84624200 1.29491800 0.39742100

C -2.38285900 1.03289000 1.84905700

H -1.60881100 1.19730600 2.60678200

H -3.16047900 1.78388300 2.05883800

H -2.83828400 0.05180800 1.99985300

C -0.88761900 2.50540800 0.08918100

H -0.04996900 2.19869100 -0.54841800

H -1.44067500 3.25718100 -0.49305300

H -0.48601000 3.00527900 0.97505300

O 2.12802400 0.15495200 -0.66990500

C 4.41331800 0.62546700 -1.18812000

H 4.00205200 1.15887900 -2.04351500

H 5.00417800 1.31213900 -0.57260100

H 5.09491700 -0.15913900 -1.53326200

C 2.82816400 -1.31448600 1.66726100

H 1.76520700 -1.22235300 1.47109900

H 3.13565000 -1.87191000 2.54433600

C 3.71660400 -0.75322300 0.84623200

H 4.78249200 -0.83885200 1.03769100

C -3.58886300 -0.30568300 -0.70841200

H -4.53536800 0.21151300 -0.79685400

C -3.41450600 -1.61394000 -0.39637500

H -4.19830100 -2.33317300 -0.19367500

C -1.99049400 -1.90604700 -0.36434300

H -1.56438300 -2.87893500 -0.15166500

C -1.29090200 -0.78108900 -0.66418400

H -0.21660300 -0.67787100 -0.73844000

Name: 2-3

Absolute *E* (Hartree): −565.294258

Absolute *G* (Hartree): −565.358037

Coordinates:

X Y Z

C 2.39199200 0.25607000 -0.00028000

H 3.39698500 0.73250500 0.00187300

C -2.92087300 0.10687800 -0.00043400

B 1.51145600 1.59295900 0.00200700

C 1.08808800 2.24821700 -1.36026100

H 0.95026300 3.33171500 -1.30110000

H 1.73979700 2.01652500 -2.20728400

H 0.10138900 1.82295000 -1.59996400

C 1.09086400 2.24551300 1.36638200

H 0.10123500 1.82543700 1.60322200

H 1.74022000 2.00736700 2.21337500

H 0.95926200 3.32999600 1.31062200

O -1.92494600 0.79405200 0.00600400

C -1.85144000 -2.09720900 0.00986600

H -0.87503800 -1.62548900 0.01651600

H -1.88743400 -3.18029700 0.01079100

C -2.96948100 -1.37255300 0.00086800

H -3.95364100 -1.82589300 -0.00587500

C 2.29990800 -0.67377400 -1.17668000

H 2.34669200 -0.35572100 -2.20876800

C 2.14058000 -1.94146700 -0.73629900

H 2.03335500 -2.82268000 -1.35651600

C 2.13899300 -1.94449600 0.72606800

H 2.03060600 -2.82830000 1.34239900

C 2.29737600 -0.67864800 1.17210800

H 2.34199700 -0.36487300 2.20559500

O -4.16898900 0.64053600 -0.01007400

H -4.06752700 1.60407100 -0.01039000

Name: 2-4

Absolute *E* (Hartree): −564.797243

Absolute *G* (Hartree): −564.853451

Coordinates:

X Y Z

C -1.74234500 0.28845000 -0.05610500

H -2.51280400 1.05894200 -0.20735000

C 1.66737300 -0.12316100 -0.55105800

B -0.37169000 1.29994800 0.16492600

C -0.49793800 2.09810500 1.58438300

H 0.32392600 2.81967700 1.69866000

H -1.43416800 2.67069500 1.65072600

H -0.46079800 1.42971600 2.45406700

C -0.23198100 2.33934000 -1.08078700

H 0.69382800 2.92689100 -1.00071100

H -0.21724600 1.86377700 -2.06541500

H -1.06352100 3.05993700 -1.06351100

O 0.88665400 0.40038600 0.34910500

O 1.59069900 -0.02309700 -1.77154100

C -1.64556400 -0.65093900 -1.20528800

H -1.40677000 -0.34691800 -2.21526400

C -1.79134500 -1.92989200 -0.76991300

H -1.69525600 -2.82771800 -1.37087300

C -2.05677500 -1.90694200 0.66367900

H -2.19894200 -2.78542400 1.28460600

C -2.06459600 -0.61432400 1.08257300

H -2.22644100 -0.27614700 2.09821500

C 2.79642500 -0.93246900 0.03511300

H 3.45509300 -1.36829400 -0.71085300

C 3.00289800 -1.12399600 1.33701500

H 2.33083800 -0.68666700 2.06650100

H 3.83497800 -1.72155100 1.69839200

Name: 2-5

Absolute *E* (Hartree): −545.408848

Absolute *G* (Hartree): −545.473635

Coordinates:

X Y Z

C 2.26114000 0.21939700 0.03723500

H 3.26815700 0.66554700 0.19454400

C -2.17401000 -0.44419400 0.09545800

B 1.42318300 1.58453600 0.12900300

C 1.09669200 2.37377300 -1.18800100

H 0.86305100 3.42938100 -1.02508400

H 1.87370200 2.29862500 -1.95597600

H 0.19690800 1.90589600 -1.61131400

C 1.02275200 2.16086300 1.53358200

H -0.05437300 1.96814500 1.64070500

H 1.52125600 1.70784000 2.39456700

H 1.14164200 3.24812300 1.58750500

O -1.57424100 0.60893100 -0.08148700

N -1.53504100 -1.59352600 0.44969600

H -2.02504800 -2.46760300 0.53967500

H -0.52438300 -1.59069100 0.51919200

C -4.40658600 0.48154600 -0.38172400

H -3.94208300 1.44591200 -0.55579800

H -5.48203200 0.40312000 -0.48750500

C -3.65842400 -0.56683700 -0.04721900

H -4.11005900 -1.53904900 0.13137900

C 2.28212800 -0.56394200 -1.24193000

H 2.41841700 -0.12438100 -2.22013800

C 2.09484300 -1.87532000 -0.97397100

H 2.04993300 -2.67664200 -1.70054000

C 1.95674900 -2.05057200 0.47182800

H 1.81834100 -3.00329300 0.96940400

C 2.05770300 -0.84398400 1.07762900

H 2.01194300 -0.65476700 2.14106700
